# Influence of oral contrast type and volume on patient experience and quality of luminal distension at MR Enterography in Crohn’s disease: an observational study of patients recruited to the METRIC trial

**DOI:** 10.1007/s00330-022-08614-9

**Published:** 2022-03-03

**Authors:** Gauraang Bhatnagar, Sue Mallett, Laura Quinn, Rajapandian Ilangovan, Uday Patel, Asif Jaffer, Christopher Pawley, Arun Gupta, Anthony Higginson, Andrew Slater, Damian Tolan, Ian Zealley, Steve Halligan, Stuart A Taylor, on behalf of the METRIC study investigators

**Affiliations:** 1 Centre for Medical Imaging, University College London, Charles Bell House, 43-45 Foley Street, London, W1W 7TS UK; 2 Institute of Applied Health Research, NIHR Birmingham Biomedical Research Centre, College of Medical and Dental Sciences, University of Birmingham, Edgbaston, Birmingham, B15 2TT UK; 3 Intestinal Imaging Centre, St Mark’s Hospital, LNWUH NHS Trust, Harrow, UK; 4 Department of Radiology, Portsmouth Hospitals NHS Trust, Portsmouth, UK; 5 Department of Radiology, Oxford University Hospitals NHS Trust, Oxford, UK; 6 Department of Radiology, St James’s University Hospital, Leeds Teaching Hospitals NHS Trust, Beckett Street, Leeds, LS9 7TF UK; 7 Department of Radiology, Ninewells Hospital, Dundee, DD1 9SY Scotland, UK

**Keywords:** Crohn’s disease, Diagnostic imaging, Magnetic resonance imaging

## Abstract

**Objectives:**

To compare the distention quality and patient experience of oral mannitol and polyethylene glycol (PEG) for MRE.

**Methods:**

This study is a retrospective, observational study of a subset of patients enrolled in a multicentre, prospective trial evaluating the diagnostic accuracy of MRE for small bowel Crohn’s. Overall and segmental MRE small bowel distention, from 105 patients (64 F, mean age 37) was scored from 0 = poor to 4 = excellent by two experienced observers (68 [65%] mannitol and 37 [35%] PEG). Additionally, 130 patients (77 F, mean age 34) completed a questionnaire rating tolerability of various symptoms immediately and 2 days after MRE (85 [65%] receiving mannitol 45 [35%] receiving PEG). Distension was compared between agents and between those ingesting ≤ 1 L or > 1 L of mannitol using the test of proportions. Tolerability grades were collapsed into “very tolerable,” “moderately tolerable,” and “not tolerable.”

**Results:**

Per patient distension quality was similar between agents (“excellent” or “good” in 54% [37/68] versus 46% [17/37]) with mannitol and PEG respectively. Jejunal distension was significantly better with mannitol compared to PEG (40% [27/68] versus 14% [5/37] rated as excellent or good respectively). There was no significant difference according to the volume of mannitol ingested. Symptom tolerability was comparable between agents, although fullness following MRE was graded as “very tolerable” in 27% (12/45) of patients ingesting PEG, verses 44% (37/84) ingesting mannitol, difference 17% (95% CI 0.6 to 34%).

**Conclusion:**

Mannitol-based solutions and PEG generally achieve comparable distension quality and side effect profiles, although jejunal distension is better quality with mannitol. Neither distension quality nor side-effect profile is altered by ingestion of more than 1 L of mannitol.

**Key Points:**

*• Mannitol-based and PEG-based oral preparation agents generally achieve comparable distension quality for MRE with the exception of the jejunum which is better distended with mannitol.*

*• Mannitol-based and PEG-based oral preparation agents used for MRE have similar side effect profiles.*

*• Neither distension quality nor side-effect profile is altered by ingestion of more than 1 L of mannitol.*

**Supplementary Information:**

The online version contains supplementary material available at 10.1007/s00330-022-08614-9.

## Introduction

Cross-sectional imaging is sensitive and specific for diagnosing and staging small bowel Crohn’s disease (CD). Magnetic resonance enterography (MRE) has the advantage of not exposing patients to ionising radiation [1]. It relies upon the combination of good small bowel distension and multi-parametric sequences to accurately identify disease and phenotype as either predominantly inflammatory or fibrostenotic [2, 3]. Diagnostic accuracy pivots on the quality of luminal distension; poor distension can both conceal or mimic disease, leading to misdiagnosis. Distension is influenced by the type and volume of oral preparation agent ingested and a variety of protocols are used clinically [4–7]. A recent literature review by the European Society of Gastrointestinal and Abdominal Radiology (ESGAR) found no evidence for the superiority of one oral preparation over another and made no specific recommendation on either the optimal agent nor ingested volume [8].

Whilst MRE is generally well tolerated, compared to small bowel ultrasound (SBU), it is more burdensome and causes symptoms, such that SBU is usually preferred by patients [9]. Gastrointestinal effects related to the oral preparation agent are most commonly cited by patients as the least acceptable characteristic of MRE [9].

The existing literature investigating oral preparation agents has largely focused on a small number of healthy individuals at single centres [5, 10–15]. Findings may not generalise to the (often) symptomatic patients undergoing MRE. Indeed, surprisingly, few studies have investigated oral contrast agents in patients [16–17].

We conducted a prospective multi-centre study comparing the diagnostic accuracy of MRE with SBU in Crohn’s disease [Diagnostic accuracy of magnetic resonance enterography and small bowel ultrasound for the extent and activity of newly diagnosed and relapsed Crohn's disease (METRIC): a multicentre trial [1, 18]. The study afforded the pragmatic opportunity to prospectively compare two commonly used MRE oral contrast agents, mannitol and polyethylene glycol (PEG) exactly as they are employed in clinical practice and was a pre-specified secondary outcome [18, 19]. Specifically, the aims of the current study were to compare (1) distension quality and (2) patient symptoms, according to the agent ingested. We also investigated the influence of ingested volume on image distention quality and patients’ symptoms.

## Materials and methods

### Study population

This study was conducted as a pre-specified sub-study of a larger multi-centre, prospective cohort trial investigating the sensitivity of MRE and SBU (METRIC Trial). The trial recruited two patient cohorts: (1) newly diagnosed and (2) established disease, clinically suspected of luminal relapse [1, 18]. Full ethical permission was obtained (NRES Committee September 2013 reference 13/SC/0394).

The current study was a sample of convenience based on the following: (1) receipt of MRE datasets from recruitment sites for central distension scoring during the course of the trial up until October 2015, (2) available information on oral contrast agent type and volume ingested and, (3) return of completed patient experience questionnaires.

The study cohort consisted of 114 (34%) of the 335 patients recruited to the main diagnostic accuracy trial. Of these 114, 9 patients were excluded subsequently (6 did not have a diagnosis of CD and 3 withdrew from the trial). The final cohort consisted of 105 patients recruited across 6 sites. Overall, 68 (65%) received mannitol-based oral preparation and 37 (35%) received PEG-based oral preparation. A subset of 66 patients from the current study has been reported in part previously [19].

### Study design

#### Imaging protocol

The main diagnostic accuracy trial was a pragmatic trial. As such, all recruitment sites used their usual clinical protocol for all MRE examinations. There was no specific stipulation as to the type of oral preparation agent to be used or volume to be ingested.

This study included patients recruited from six of the eight centres that took part in the main METRIC trial. The remaining two centres had not provided data by the submission deadline for this substudy due to delays in commencing recruitment. Four of the six sites used a mannitol-based oral preparation regimen (two sites used mannitol 2.5% alone, one site used mannitol 2.5% and 0.2% Locust Bean Gum and one site used mannitol 2.5% and 2 scoops Carobel, Cow & Gate, Nutricia Ltd.). Two sites utilised a PEG oral preparation without additives (69 g Klean prep/litre, Helsinn-Birex pharmaceuticals Ltd.). Full details of the differing oral prep regimens employed at all sites are provided in Appendix 8. Patients were instructed to drink the provided volume of oral preparation (1.5–2 L) at a steady rate over a 45–60-min period according to tolerance and encouraged by radiography staff at regular intervals.

A minimum dataset of sequences was acquired including T2-weighted images with and without fat saturation, steady-state free precession gradient-echo images, diffusion-weighted images, and T1-weighted images after intravenous gadolinium injection (see Appendix 1 for minimal MRE dataset)*.*

#### Recording MRE oral preparation details

Recruitment sites were requested to record prospectively the exact volume of ingested contrast agent.

#### Patient experience questionnaires

Patients recruited to the main diagnostic accuracy trial were invited to complete a three-part questionnaire asking their experience of various symptoms before and after oral preparation. The questionnaire was given to participants by radiographers. Patients were asked to complete a baseline questionnaire the day of their MRE but before ingesting the oral contrast and to then complete a second questionnaire immediately after MRE. These were then handed to radiography staff. The third questionnaire was completed two days later to capture symptoms for 48 h post-MRE. Patients were asked to return this either by hand or mail (stamped, addressed envelopes were provided). At each of the three time points, participants were asked to rate tolerability (“not at all tolerable,” “somewhat tolerable,” “moderately tolerable,” “very tolerable”) and record symptoms of fullness, regurgitation, vomiting, abdominal pains/spasms, and diarrhoea.

The questionnaire is reproduced in Appendix 2.

#### Grading of bowel distension

All MRE examinations were anonymised (for patient- and site-specific information) and uploaded to an online viewing platform (Biotronics 3Dnet, Biotronics 3D).

Two consultant gastrointestinal radiologists reporting MRE as part of their routine clinical work at a tertiary referral centre for 10 and 4 years, respectively, reviewed all MREs independent of each other and performed qualitative distension grading in isolation. Observers were blinded to the oral contrast agent used and its volume.

The small bowel was divided into the duodenum, jejunum, ileum, and terminal ileum. The terminal ileum was defined as the terminal 10 cm of small bowel. The jejunum was defined as the proximal bowel lying largely to the left of a diagonal line drawn from the right lower quadrant to the left lower quadrant, demonstrating a typical “feathery” fold pattern, and the ileum as the bowel interposed between the jejunum and terminal ileum [18]. Right colonic segments (caecum, ascending colon, and transverse colon) were defined as described previously [20].

Segmental distension was graded qualitatively using the methods described by Saini et al [21]. Each small bowel and right colonic segment (caecum, ascending colon, transverse colon) was graded independently by each observer on a 5-point scale: 0, very poor distension; 1, poor distension; 2, fair distension; 3, good distension; and 4, excellent distension [5, 7, 15, 21]. The reviewers were instructed to use the entire image set as part of their assessment.

Observers also scored the overall per-patient quality of small bowel distension using the same scale. Observers were free to use all MRE sequences when making their grading decision.

### Statistical analysis

The frequency of “excellent” or “good” distension according to the type of oral contrast was calculated on a per-patient level and for individual intestinal segments. In cases of reader disagreement, the best distension score of the two was used for the main analysis but results from each individual reader are also presented. Per-patient and segmental distension scores were compared between the two oral contrast groups using the test of proportions. Distension scores were also compared according to the volume of mannitol ingested (1 L or less vs. more than 1 L). The distribution of data for the volume of PEG ingested was insufficient to undertake meaningful analysis for this agent.

For the purposes of analysis, tolerability grades were collapsed as follows: (1) very tolerable (“I did not experience this symptom” and “very tolerable”), (2) moderately tolerable (combining “moderately” and “somewhat” tolerable), and (3) not tolerable (“not at all tolerable”) and compared according to oral contrast agent and volume of mannitol ingested (1 L or less vs. more than 1 L).

Inter-observer variability for distension scores were analysed using Gwet’s chance–adjusted agreement coefficient [22]. Strength of agreement was interpreted using the Landis and Koch criteria [23]: < 0.00 = poor, 0.00–0.20 = slight, 0.21–0.40 = fair, 0.41–0.60 = moderate, 0.61–0.80 = substantial, and 0.81–1.00 = excellent.

## Results

### Distension 

#### Patient characteristics

Detailed patient characteristics are provided in Appendix 3. Importantly there were no important differences between the groups in potential confounders which could influence distension, notably presence of stenosis, prior resection, and disease activity (as measured by the HBI and CRP).

The volumes of mannitol- and PEG-based oral preparation ingested are presented in Appendix 4. Of the 68 patients who ingested mannitol, 3 did not have information on volume ingested (and so were excluded from volume analysis). Overall, patients ingested between 200 mL and 1.8 L, and 49% (32/65) ingested more than 1 L. Of the 37 patients who ingested PEG, the volume ranged between 300mL and 1.5 L, and 11% (4/35) of patients drank more than 1 L. Overall, 34% (22/65) ingested exactly 1 L of mannitol and 35% (13/37) ingested exactly 1 L of PEG.

#### Qualitative distension assessment

##### Quality of luminal distension according to mannitol or PEG oral preparation

Using the best distension score of the two readers, there was no significant difference in the proportion of patients achieving excellent or good distention between mannitol or PEG-based preparations. Specifically, per-patient distension with mannitol-based preparation was rated as excellent or good in 54% (37/68) versus 46% (17/37) with PEG-based preparation (percentage difference [95% CI] 8 [−11 to 28]) (Table 1).

**Table 1:** Number of patients\segments achieving good (or excellent) distension according to oral contrast agent

	Number of patients/segments where distension was graded as excellent/good by at least one observer		
	Mannitol (*n *= 68) *n*/N* (%)	Polyethylene glycol (PEG) (*n *= 37) n/N* (%)	Difference between contrasts** % (95% CI)
Patients	37/68 (54)	17/37 (46)	8 (−11 to 28)
Segments			
Duodenum	11/68 (16)	5/37 (14)	2 (−11 to 17)
Jejunum	27/68 (40)	5/37 (14)	26 (10 to 42) *p *= 0.0053
Ileum	51/68 (75)	28/37 (76)	-1 (−18 to 17)
Terminal Ileum	37/68 (54)	20/37 (54)	0 (−20 to 20)
Caecum	22/51 (43)	14/28 (50)	-7 (−30 to 16)
Ascending colon	43/61 (70)	23/33 (70)	0 (−19 to 20)
Transverse colon	41/64 (64)	16/37 (43)	20 (0 to 41) *p *= 0.0420

*In some patients, segments had been excised and so could not be assessed

**Mannitol minus polyethylene glycol (PEG)

At a segmental level, ileal distension quality was greater for both preparations, followed by the terminal ileum and the jejunum (Table 1). Jejunal and transverse colon segmental distension were significantly better distended in the mannitol group in comparison to the PEG group (Table 1). This pattern was consistent for both individual readers, reaching statistical significance for reader 2 in the jejunum (95% CI of difference in distension 11 to 37%) and reader 1 in the transverse colon (95% CI of difference in distension 5 to 44%) (Appendix 9).

In general, the distension quality grading was comparable across both individual readers with the exception of the TI (Appendix 9). Reader 1 graded a greater proportion of TI segments as good/excellent distension on both preparations (mannitol 36 (53%) and PEG 15 (41%) versus reader 2 (mannitol 20 (29%) and PEG (24%)).

#### Overall Inter-observer variability

Table 2 demonstrates inter-reader agreement for patients/segments where the two readers rated distention as “excellent” or “good”. On a per-patient basis, there was substantial agreement between readers, with reader 1 rating 45% (48/105) and reader 2 rating 42% (45/102) of MREs as achieving “excellent” or “good” distention. There was excellent inter-reader agreement in assessing duodenal distension (Gwet’s AC = 0.84 Gwet’s AC 0.84 (10% and 8% assessed good/excellent distension by the two readers)) but only fair for terminal ileal distension (Gwet’s AC = 0.40 (49% and 28% assessed good/excellent distension by the two readers)). There was substantial agreement in assessing jejunal distension (Gwet’s AC = 0.68 assessed as good to excellent distension in only 20 and 21% by the two readers). There was a moderate agreement for the ileum (Gwet’s AC = 0.49 (59% and 65% assessed good/excellent distension by the two readers)).

**Table 2:** Excellent or good distension overall and by segment for the two readers

		Reader 1	Reader 2	Agreement	**Gwet’s AC**
Segment	N*	***N*(%)**	***N*(%)**	**% (95% CI)**	
Overall	105	48 (45)	45 (42)	82 (75 to 90)	0.65
Duodenum	105	11 (10)	9 (8)	87 (80 to 93)	0.84
Jejunum	105	21 (20)	22 (21)	79 (71 to 86)	0.68
Ileum	105	63 (59)	70 (65)	73 (64 to 81)	0.49
Terminal_ileum	105	52 (49)	30 (28)	68 (59 to 77)	0.40
Caecum	80	30 (37)	31 (39)	84 (75 to 92)	0.69
Ascending_C	94	61 (64)	53 (55)	77 (69 to 86)	0.56
Transverse_C	101	53 (51)	33 (32)	69 (60 to 78)	0.40

*In some patients, segments had been excised and so could not be assessed

##### Impact of oral mannitol volume ingested

There was no significant difference in distension quality either overall or on a segmental basis according to the volume of mannitol ingested (1 L or less vs more than 1 L) (Table 3). Ileal distension quality was most frequently rated the greatest for both cohorts. The jejunum achieved good/adequate distension in 15/32 (47%) ingesting more than 1 L of mannitol versus 11/33 (33%) of patients ingesting 1 L or less but this difference was not of statistical significance (−14% (−37 to 10)). Of note, the sample size was limited and therefore a test of proportions was not appropriate.

**Table 3:** Number of patients/segments achieving good or excellent distension according to mannitol oral contrast volume ingested

	Volume less or equal than 1L N* (%)	Volume more than 1L N* (%)	Difference** % (95% CI)
Patients	17/33 (52)	18/32 (56)	−5 (−29 to 19)
Segments			
Duodenum	5/33 (15)	5/32 (16)	
Jejunum	11/33 (33)	15/32 (47)	−14 (−37 to 10)
Ileum	24/33 (73)	25/32 (78)	
Terminal_ileum	17/33 (52)	19/32 (59)	
Caecum	8/19 (42)	12/30 (40)	
Ascending_C	18/27 (67)	23/31 (74)	
Transverse_C	17/29 (59)	23/32 (72)	−13 (−37 to 10)

*In some patients, segments had been excised and so could not be assessed

**Volume less or equal than 1L vs volume more than 1L (the statistical power is limited and therefore unable to show a statistical difference between proportions)

### Patient symptoms

Overall, 143 (43%) of the 335 patients recruited to the main diagnostic accuracy trial had information on oral contrast type and volume ingested as well as providing a completed questionnaire immediately after MRE. Of these, 13 patients were excluded (10 patients did not have a diagnosis of CD and 3 withdrew from the trial). The final cohort consisted of 130 patients recruited across 6 sites, with 85 (65%) receiving mannitol-based oral preparation and 45 (35%) receiving PEG-based oral preparation. Full patient characteristics are provided in Appendix 5. 78 (60%) of these 130 patients were also part of the cohort included in the qualitative distension study.

The delayed patient symptom questionnaire (for symptoms over the 2-days following MRE) had a variable return rate: mannitol 44/85 (52%) and PEG 44/45 (98%). All patients recording any symptom as “not tolerable” immediately after MRE completed the delayed symptom questionnaire.

The volumes of mannitol- and PEG-based oral preparation ingested are shown in Appendix 6. The volume of mannitol ingested ranged between 200 mL and 1.8 L with 34% (29/85) drinking 1 L. The volume PEG ingested ranged between 300mL and 1.5 L, with 33% (15/45) drinking 1 L.

#### Patient symptoms according to mannitol or PEG oral preparation

Baseline symptoms were comparable between mannitol and PEG groups (Appendix 7).

**Fig. 1:**
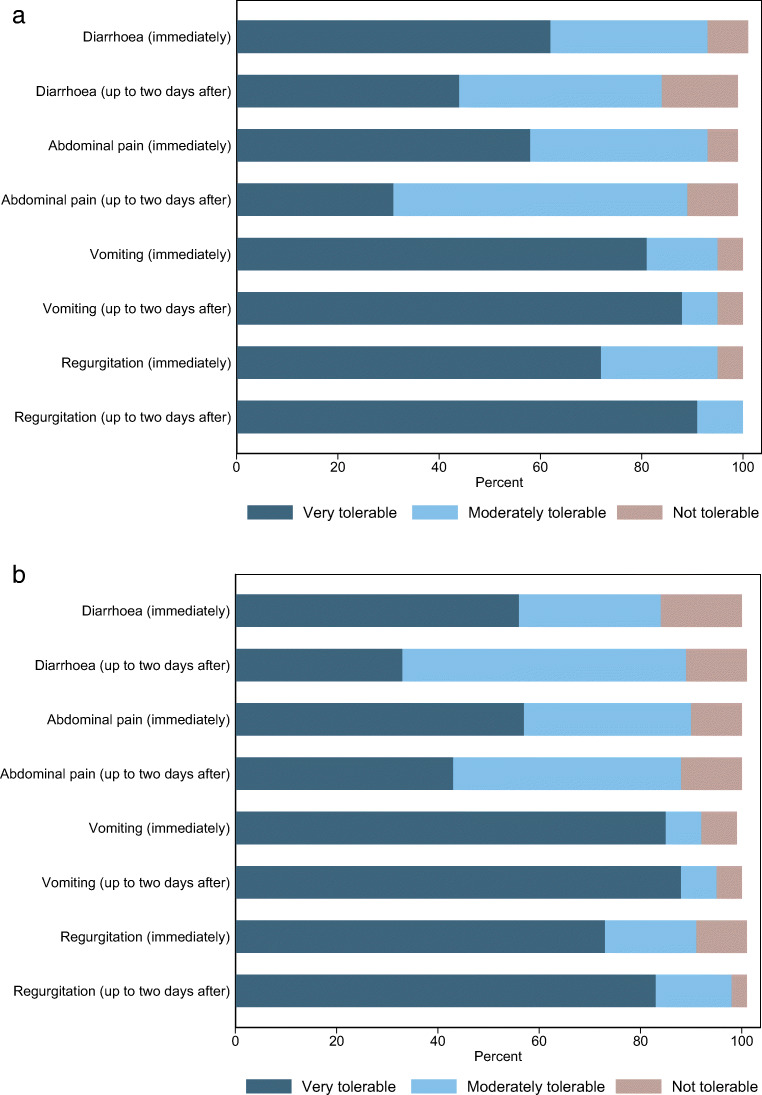
Comparison of patient symptoms dependent on oral preparation ((**a**) mannitol-based and (**b**) PEG-based) immediately after and up to 2 days after the MRE

Tolerability of symptoms immediately after MRE and over the next 2 days is shown in Table 4 and graphically in Fig. 1.

**Table 4:** Patient symptoms by contrast agent and time point following MRE

	Mannitol (*N *= 85)			Polyethylene Glycol (PEG) (*N *= 45)		
	Very tolerable	Moderately tolerable	Not tolerable	Very tolerable	Moderately tolerable	Not tolerable
	*n*/*N*^a^ (%)	*n*/*N*^a^ (%)	*n*/*N*^a^ (%)	*n*/*N*^a^ (%)	*n*/*N*^a^ (%)	*n*/*N*^a^ (%)
Symptoms immediately following MRE						
A feeling of fullness	37/84 (44*)	46/84 (55)	1/84 (1)	12/45 (27*)	32/45 (71)	1/45 (2)
Regurgitation	62/83 (75)	18/83 (22)	3/83 (4)	31/43 (72)	8/43 (19)	4/43 (9)
Vomiting	72/81 (89)	7/81 (9)	2/81 (2)	38/44 (86)	3/44 (7)	3/44 (7)
Abdominal pain/spasms	50/82 (61)	28/82 (34)	4/82 (5)	25/43 (58)	14/43 (33)	4/43 (9)
Diarrhoea	54/82 (66)	24/82 (29)	4/82 (5)	25/44 (57)	12/44 (27)	7/44 (16)
Symptoms for the 2 days following MRE						
Flatulence	23/48 (48)	20/48 (42)	5/48 (10)	22/42 (52)	15/42 (36)	5/42 (12)
Regurgitation	39/43 (91)	4/43 (9)	0/43 (0)	33/41 (80)	6/41 (15)	2/41 (5)
Vomiting	39/44 (89)	3/44 (7)	2/44 (5)	36/41 (88)	3/41 (7)	2/41 (5)
Abdominal pain/spasms	15/49 (31)	29/49 (59)	5/49 (10)	18/42 (43)	19/42 (45)	5/42 (12)
Diarrhoea	24/55 (44)	22/55 (40)	9/55 (16)	14/44 (32)	24/44 (55)	6/44 (14)

^a^The number of patients answering each survey question varies, so numbers and percentages are given for those responding

*The only significant difference in experience between patients receiving mannitol and those receiving PEG was in the feeling of fullness immediately after contrast, where 17% more patients (95% CI 0.6 to 34%) had a very tolerable feeling of fullness with mannitol compared to PEG

In general, symptom tolerability immediately after the MRE was comparable between the two preparations. For the mannitol group, vomiting and regurgitation were the best-tolerated symptoms and abdominal pain the least. Symptoms of fullness were graded “very tolerable” in just 27% (12/45) of patients ingesting PEG, a significantly lower proportion than for mannitol (44% [37/84]), a 17% difference (95% CI 0.6–34%)

For both preparations, tolerability of abdominal pain and diarrhoea was generally rated worse after 2 days than immediately after MRE, and again largely comparable between preparations. For example, 2 days post-MRE, patients reported abdominal pain as very tolerable in 31% (15/49) and 43% (18/42) after ingesting mannitol and PEG respectively, a decrease from 61% (50/82) and 58% (25/43) immediately after MRE. Similarly, diarrhoea was worse after 2 days for both preparations; for example, 32% (14/44) reported it as very tolerable 2 days after PEG ingestion compared to 57% (25/44) immediately after MRE. Regurgitation improved after 2 days.

#### Patient symptoms depending on volume of oral preparation ingested

The influence of ingested mannitol volume on patient symptoms is shown in Table 5. The response rate for the delayed symptom questionnaire was 30/40 (75%) and 25/45 [(56%) for the “1 L or less” and “more than 1 L” groups respectively.

**Table 5:** Patient experience according to mannitol oral contrast volume ingested

	Volume less or equal than 1L (*N*=40)			Volume more than 1L (*N*=45)		
	Very tolerable	Moderately tolerable	Not tolerable	Very tolerable	Moderately tolerable	Not tolerable
	*n/N*^a^ (%)	*n/N*^a^ (%)	*n/N*^a^ (%)	*n/N*^a^ (%)	*n/N*^a^ (%)	*n/N*^a^ (%)
Symptoms immediately following MRE						
A feeling of fullness	18/39 (46)	20/39 (51)	1/39 (3)	19/45 (42)	26/45 (58)	0/45 (0)
Regurgitation	30/40 (75)	10/40 (25)	0/40 (0)	32/43 (74)	8/43 (19)	3/43 (7)
Vomiting	34/38 (89)	4/38 (11)	0/38 (0)	38/43 (88)	3/43 (7)	2/43 (5)
Abdominal pain/spasms	25/39 (64)	13/39 (33)	1/39 (3)	25/43 (58)	15/43 (35)	3/43 (7)
Diarrhoea	23/39 (59)	14/39 (36)	2/39 (5)	31/43 (72)	10/43 (23)	2/43 (5)
Symptoms up to 2 days following MRE						
Flatulence	14/26 (54)	12/26 (46)	0/26 (0)	9/22 (41)	8/22 (36)	5/22 (23)
Regurgitation	23/24 (96)	1/24 (4)	0/24 (0)	16/19 (84)	3/19 (16)	0/19 (0)
Vomiting	23/25 (92)	2/25 (8)	0/25 (0)	16/19 (84)	1/19 (5)	2/19 (11)
Abdominal pain/spasms	9/26 (35)	14/26 (54)	3/26 (12)	6/23 (26)	15/23 (65)	2/23 (9)
Diarrhoea	14/30 (47)	11/30 (37)	5/30 (17)	10/25 (40)	11/25 (44)	4/25 (16)

^a^The number of patients answering each survey question varies, so numbers and percentages are given for those responding

In general, symptoms immediately after MRE were comparable between those ingesting 1 L or less compared to those ingesting more than 1 L.

Diarrhoea immediately after MRE was rated “very tolerable” by 59% (23/39) of patients drinking 1 L or less and 72% (31/43) of patients drinking more than 1 L; not statistically significant.

Similarly, symptoms up to 2 days after MRE were comparable between the two volumes ingested. For example, abdominal pain/spasms were “very tolerable” in 35% (9/26) of patients drinking 1 L or less and 26% (6/23) of patients drinking more than 1 L. Diarrhoea was “very tolerable” in 47% (14/30) of patients drinking 1 L or less and 40% (10/25) of patients drinking more than 1 L.

The same pattern of worsening tolerability of abdominal pain and diarrhoea but improved regurgitation after 2 days was observed in both volume groups.

## Discussion

We conducted a large multi-centre, prospective diagnostic accuracy trial investigating the sensitivity of MRE and SBU (METRIC trial) [1]. This afforded the opportunity to prospectively assess the quality of bowel distension achieved in representative clinical practice by two of the most commonly used MRE distention agents, and to compare symptoms following ingestion. The results may also translate to other luminal investigations requiring luminal distension such as CT enterography and hydrosonography.

To date, the majority of previous literature pertaining to oral contrast agents has reported healthy volunteers who are unlikely to represent patients commonly undergoing MRE [5, 10–15], or limited to retrospective studies of small numbers at single centres [21, 24, 25]. In this regard, our work adds to the current literature.

We found that, overall, there were no major differences in distention quality between either mannitol-based preparations or PEG. However, we did find some evidence that whilst jejunal distension remains challenging, it is more commonly good or excellent quality with mannitol (40%) compared to PEG (14%). This is potentially an important observation given difficulties with jejunal distention during MRE (as opposed to MR enteroclysis) and the potential impact on diagnostic accuracy. Importantly, the two groups were generally well-matched in terms of presentation (new diagnosis versus relapse), presence of stenosis, and history of prior surgical resection, which increases our confidence that our findings are real and not secondary to unequal disease phenotypes across cohorts. Although the colon is not the primary target for MRE, it is interesting to note superior transverse colonic distension with mannitol.

We also found ingesting more than 1 L of mannitol did not confer any beneficial effect. This concurs with Ajaj et al who reported that in a study of 10 volunteers, 1000, 1200, and 1500 mL of mannitol all gave similar quality distension [5]. Overall, our data suggest there is no need for patients to ingest more than 1 L of oral contrast. Perhaps surprisingly, we did not find any difference in the immediate or delayed symptoms experienced by patients, regardless of the volume of contrast. There was perhaps a trend for greater diarrhoea in those drinking more contrast, but this was not statistically significant perhaps due to underpowering.

We also found mannitol and PEG were similarly tolerable, although patients ingesting PEG reported that fullness was significantly less tolerable immediately after MRE compared to those ingesting mannitol. We note that the proportion of patients ingesting 1 L or more was lower in the PEG cohort than in the mannitol cohort. Whilst the exact reasons for this observation are uncertain, it is possible the greater feelings of fullness in the PEG group led to reduced overall intake.

Of note, abdominal pain and diarrhoea increased over the 2 days after MRE compared to immediately afterwards. This is perhaps unsurprising as it takes time for contrast to traverse through the gut and concurs with a recent study in which oral contrast was rated the most unpleasant component of MRE; 18% of patients take longer than 1 day to recover [9]. Patients should be warned of this prior to MRE.

There are no published studies comparing mannitol and PEG in MRE but a recently published randomised controlled trial did compare the two preparations in 70 patients undergoing CT enterography at a single centre. Each patient underwent 2 L of PEG bowel preparation prior to ingesting either 1.5 L of mannitol or PEG solution. The study reported no significant differences in the quality of luminal distension between the agents but stated that patients undergoing mannitol preparation reported nausea as more tolerable, the taste as more acceptable, and were more willing to ingest again compared to patients undergoing PEG preparation. [26]

Our study has limitations. We investigated the impact of mannitol and PEG-based oral preparations alone, as these were the two agents utilised at centres recruiting to the main diagnostic accuracy trial. The number of patients undergoing PEG-based oral preparation was smaller, and as a result, we were unable to incorporate the PEG cohort into the assessment of the impact of ingested oral volume on either luminal distension or patient experience. Furthermore, whilst we had a good proportion of delayed patient experience questionnaires returned in the PEG cohort, this was much reduced in the mannitol cohort, which impairs comparison for delayed symptoms. This may in part reflect the different tenacity of individual recruitment sites when encouraging patients to return questionnaires but risks some bias. Whilst we compare the main agents of the oral preparation, we acknowledge that some mannitol preparations utilised additives such as LBG or Carobel in small quantities and we have not assessed the specific contribution of these additives. Both readers for the qualitative assessment of luminal distension work at one centre which employed a mannitol-based preparation. This may introduce some bias related to their prior experience. For practical reasons, other centres did not provide readers for this substudy although that would have been optimal. Agreement between readers was generally good, although less so for the terminal ileum in particular. Reassuringly though, both readers were consistent in the relative grading of distension quality between the two preparations for all segments so any disagreement in absolute levels of distension did not impact our main conclusion. Whilst the main trial evaluated the diagnostic accuracy of MRE (and SBU), this substudy was underpowered to draw conclusions on whether the differing quality of luminal distension affected the overall diagnostic accuracy. This would be a useful topic for further research. However, it is reassuring that although distension quality was judged as excellent in slightly over 50% of terminal ileal segments, the results on the main trial showed MRE has a high sensitivity for terminal ileal Crohn’s disease, suggesting accurate diagnosis does not always require optimal distension. Anecdotally, the segmental small bowel distension will alter throughout the MRI acquisition (as the study typically takes 30–45 min to acquire); it would be of interest to review whether this change in segmental distension is different for differing luminal preparation agents; this was felt to be outside the remit of this study.

Patients who reported at least one symptom as “not tolerable” generally completed the day 2 questionnaire, whereas those less affected completed fewer. This may introduce spectrum bias, with a greater proportion returning this questionnaire more likely to experience less tolerable symptoms. Ours was a convenience sample based on data return, which could induce bias. Reassuringly, we found no major difference between PEG and mannitol cohorts regarding disease phenotype or baseline symptom level. Although to our knowledge, ours is the largest prospective patient study on this topic to date, we did not perform a prior power calculation and so some of our comparisons are likely unpowered, for example, the effects of agent volume on distension and symptoms.

In summary, mannitol-based solutions and PEG generally achieve comparable distension quality and side effect profiles, although jejunal distension is more frequently of better quality with mannitol. Distension quality is not improved by ingestion of more than 1 L, although doing so does not adversely influence patent tolerability.

## Supplementary information

ESM 1 (DOCX 86 kb)

ESM 2 (PDF 125 kb)

## Abbreviations

CD: Crohn’s disease
ESGAR: European Society of Gastrointestinal and Abdominal Radiology
METRIC Trial: MR Enterography or ulTRasound In Crohn’s disease
MRE: Magnetic resonance enterography
PEG: Polyethylene Glycol
SBU: Small bowel ultrasound

## Declarations

### List of METRIC study investigators

Rachel Baldwin-Cleland, Richard Beable, Margaret Betts, Stuart Bloom, Helen Bungay, Peter J Hamlin, Ailsa L Hart, Antony, Ilan Jacobs, Sara McCartney, Anne Miles, Charles D Murray, Richard C Pollok, Shonit Punwani, Manuel Rodriguez-Justo, Zainib Shabir, Simon Travis, Alastair Windsor, Peter Wylie, Jade Dyer, Pranitha Veeramalla, Sue Tebbs, Steve Hibbert, Richard Ellis, Fergus Thursby-Pelham, Nicola Gibbons, Claire Ward, Anthony O'Connor, Hannah Lambie, Nigel Scott, Roger Lapham, Doris Quartey, Deborah Scrimshaw, Simona Fourie, Niall Power, Phillip Lung, Ian Johnston, Mani Naghibi, Morgan Moorghen, Adriana Martinez, Christopher Alexakis, Anisur Rahman, Jonny Vlahos, Anita Wale, Teresita Beeston, Wivijin Piga, Joey Clemente, Farooq Rahman, Simona de Caro, Shameer Mehta, Roser Vega, Roman Jastrub, Hameed Rafiee, Mairead Tennent, Caron Innes, Craig Mowat, Gillian Duncan, Rebecca Greenhalgh, Rachel Hyland, Hannah Lambie, Evgenia Mainta, James Pilcher, Andrew Plumb, François Porté, Harbir Sidhu, Florian Tomini, and Steve Morris

### Guarantor

The scientific guarantor of this publication is Professor Stuart A Taylor.

### Statistics and biometry

Professor Sue Mallett kindly provided statistical advice for this manuscript.

### Study subjects or cohorts overlap

A subset of 66 patients from this study have been previously reported in Taylor SA, Mallett S, Bhatnagar G, et al (2019) Magnetic resonance enterography compared with ultrasonography in newly diagnosed and relapsing Crohn’s disease patients: the METRIC diagnostic accuracy study. *Health Technol Assess* (Rockv) 23:vii–161. doi: 10.3310/hta23420.

### Methodology

• prospective

• observational

• multicentre study

## References

[1] Taylor SA, Mallett S, Bhatnagar G et al (2018) Diagnostic accuracy of magnetic resonance enterography and small bowel ultrasound for the extent and activity of newly diagnosed and relapsed Crohn ’ s disease ( METRIC ): a multicentre trial. Lancet Gastroenterol Hepatol 3:548–558. 10.1016/S2468-1253(18)30161-4 10.1016/S2468-1253(18)30161-4 PMC6278907 29914843

[2] Jesuratnam-Nielsen K, Løgager VB, Rezanavaz-Gheshlagh B, Munkholm P, Thomsen HS (2015) Plain magnetic resonance imaging as an alternative in evaluating inflammation and bowel damage in inflammatory bowel disease - a prospective comparison with conventional magnetic resonance follow-through. Scand J Gastroenterol. 10.3109/00365521.2014.1003398 10.3109/00365521.2014.1003398 25592192

[3] Bruining DH, Bhatnagar G, Rimola J, Taylor S, Zimmermann EM, Fletcher JG (2015) CT and MR enterography in Crohn’s disease: current and future applications. Abdom Imaging. 10.1007/s00261-015-0360-9 10.1007/s00261-015-0360-9 25637127

[4] Laghi A, Paolantonio P, Iafrate F et al (2003) MR of the small bowel with a biphasic oral contrast agent (polyethylene glycol): technical aspects and findings in patients affected by Crohn’s disease. Radiol Med 106:18–27 12951547

[5] Ajaj W, Goehde SC, Schneemann H, Ruehm SG, Debatin JF, Lauenstein TC (2004) Dose optimization of mannitol solution for small bowel distension in MRI. J Magn Reson Imaging 20:648–653. 10.1002/jmri.20166 10.1002/jmri.20166 15390230

[6] Maccioni F, Viscido A, Marini M, Caprilli R (2002) MRI evaluation of Crohn’s disease of the small and large bowel with the use of negative superparamagnetic oral contrast agents. Abdom Imaging. 10.1007/s00261-001-0119-3 10.1007/s00261-001-0119-3 12066236

[7] Ajaj W, Goehde SC, Schneemann H, Ruehm SG, Debatin JF, Lauenstein TC (2004) Oral contrast agent for small bowel MRI: comparison of different additives to optimize bowel distension. Eur Radiol. 10.1007/s00330-003-2177-0 10.1007/s00330-003-2177-0 14634782

[8] Taylor SA, Avni F, Cronin CG, et al (2017) The first joint ESGAR/ ESPR consensus statement on the technical performance of cross-sectional small bowel and colonic imaging. Eur Radiol 27:2570–2582. 10.1007/s00330-016-4615-9 10.1007/s00330-016-4615-9 PMC5408044 27757521

[9] Miles A, Bhatnagar G, Halligan S et al (2018) Magnetic resonance enterography, small bowel ultrasound and colonoscopy to diagnose and stage Crohn’s disease: patient acceptability and perceived burden. Eur Radiol 43–45. 10.1007/s00330-018-5661-2 10.1007/s00330-018-5661-2 PMC6510862 30128615

[10] Young BM, Fletcher JG, Booya F et al (2008) Head-to-head comparison of oral contrast agents for cross-sectional enterography: small bowel distention, timing, and side effects. J Comput Assist Tomogr 32:32–38. 10.1097/RCT.0b013e318061961d 10.1097/RCT.0b013e318061961d 18303285

[11] Sood RR, Joubert I, Franklin H, Doyle T, Lomas DJ (2002) Small bowel MRI: Comparison of a polyethylene glycol preparation and water as oral contrast media. J Magn Reson Imaging 15:401–408. 10.1002/jmri.10090 10.1002/jmri.10090 11948829

[12] Lauenstein TC, Schneemann H, Vogt FM, Herborn CU, Ruhm SG, Debatin JF (2003) Optimization of Oral Contrast Agents for MR Imaging of the Small Bowel 1. Radiology 228:279–283 10.1148/radiol.2281020161 12750457

[13] Kuehle CA, Ajaj W, Ladd SC, Massing S, Barkhausen J, Lauenstein TC (2006) Hydro-MRI of the small bowel: effect of contrast volume, timing of contrast administration, and data acquisition on bowel distention. AJR Am J Roentgenol 187:W375–W385. 10.2214/AJR.05.1079 10.2214/AJR.05.1079 16985108

[14] Kinner S, Kuehle CA, Herbig S et al (2008) MRI of the small bowel: Can sufficient bowel distension be achieved with small volumes of oral contrast? Eur Radiol 18:2542–2548. 10.1007/s00330-008-1041-7 10.1007/s00330-008-1041-7 18500525

[15] Ajaj W, Goyen M, Schneemann H et al (2005) Oral contrast agents for small bowel distension in MRI: influence of the osmolarity for small bowel distention. Eur Radiol 15:1400–1406. 10.1007/s00330-005-2711-3 10.1007/s00330-005-2711-3 15754160

[16] Absah I, Bruining DH, Matsumoto JM et al (2012) MR enterography in pediatric inflammatory bowel disease: retrospective assessment of patient tolerance, image quality, and initial performance estimates. AJR Am J Roentgenol 199:367–375. 10.2214/AJR.11.8363 10.2214/AJR.11.8363 22915428

[17] Gottumukkala RV, LaPointe A, Sargent D, Gee MS (2019) Comparison of three oral contrast preparations for magnetic resonance enterography in pediatric patients with known or suspected Crohn disease: a prospective randomised trial. Pediatr Radiol. 10.1007/s00247-019-04378-5 10.1007/s00247-019-04378-5 30852650

[18] Taylor S, Mallett S, Bhatnagar G et al (2014) METRIC (MREnterography or ulTRasound in Crohn’s disease): a study protocol for a multicentre, non-randomised, single-arm, prospective comparison study of magnetic resonance enterography and small bowel ultrasound compared to a reference standard in those. BMC Gastroenterol 14:142. 10.1186/1471-230X-14-142 10.1186/1471-230X-14-142 PMC4134460 25110044

[19] Taylor SA, Mallett S, Bhatnagar G et al (2019) Magnetic resonance enterography compared with ultrasonography in newly diagnosed and relapsing crohn’s disease patients: the METRIC diagnostic accuracy study. Health Technol Assess 23:vii–161. 10.3310/hta23420 10.3310/hta23420 PMC6732715 31432777

[20] Taylor SA, Halligan S, Goh V et al (2003) Optimizing colonic distention for multi – detector row CT colonography : effect of hyoscine butylbromide and rectal balloon catheter 1. Radiology 229:99–108 10.1148/radiol.2291021151 12944595

[21] Saini S, Colak E, Anthwal S, Vlachou PA, Raikhlin A, Kirpalani A (2014) Comparison of 3% sorbitol vs psyllium fibre as oral contrast agents in MR enterography. Br J Radiol. 10.1259/bjr.20140100 10.1259/bjr.20140100 PMC4170861 25062448

[22] Gwet K (2014) Handbook of inter-rater reliability fourth edition.

[23] Landis J, Koch G (1977) The measurement of observer agreement for categorical data. Biometrics Mar:159–174 843571

[24] Bekendam MIJ, Puylaert CAJ, Phoa SKSS, Nio CY, Stoker J (2017) Shortened oral contrast preparation for improved small bowel distension at MR enterography. Abdom Radiol (NY). 10.1007/s00261-017-1133-4 10.1007/s00261-017-1133-4 PMC5556127 28393302

[25] Wong J, Moore H, Roger M, McKee C (2016). CT enterography: mannitol versus VoLumen. J Med Imaging Radiat Oncol.

[26] Zheng MQ, Zeng QS, Yu YQ et al (2020) Evaluation of the performance of two neutral oral contrast agents in computed tomography enterography: a randomised controlled trial. J Dig Dis 21:112–119. 10.1111/1751-2980.12835 10.1111/1751-2980.12835 PMC7065060 31825554

