# Supplementary material for: Influence of oral contrast type and volume on patient experience and quality of luminal distension at MR Enterography in Crohn’s disease: an observational study of patients recruited to the METRIC trial
Source: Eur Radiol. 2022 Mar 3;32(8):5075–85. doi: 10.1007/s00330-022-08614-9 (PMC9279188; doi:10.1007/s00330-022-08614-9)
Supplement: Supplementary file 1 — (DOCX 86 kb) [file 330_2022_8614_MOESM1_ESM.docx]

## Table 1: Number of patients\segments achieving good (or excellent) distension according to oral contrast agent

|  | **Number of patients/segments where distension was graded as excellent/good by at least one observer** | |  |
| --- | --- | --- | --- |
|  | **Mannitol (n=68)**  **n/N* (%)** | **Polyethylene Glycol (PEG) (n=37)**  **n/N* (%)** | **Difference between contrasts****  **% (95% CI)** |
| Patients | 37/68 (54) | 17/37 (46) | 8 (-11 to 28) |
| Segments |  |  |  |
| Duodenum | 11/68 (16) | 5/37 (14) | 2 (-11 to 17) |
| Jejunum | 27/68 (40) | 5/37 (14) | 26 (10 to 42)  p=0.0053 |
| Ileum | 51/68 (75) | 28/37 (76) | -1 (-18 to 17) |
| Terminal Ileum | 37/68 (54) | 20/37 (54) | 0 (-20 to 20) |
| Caecum | 22/51 (43) | 14/28 (50) | -7 (-30 to 16) |
| Ascending colon | 43/61 (70) | 23/33 (70) | 0 (-19 to 20) |
| Transverse colon | 41/64 (64) | 16/37 (43) | 20 (0 to 41)  p=0.0420 |

*In some patients segments had been excised and so could not be assessed.

** Mannitol minus Polyethylene Glycol (PEG)

## Table 2: Number of patients/segments achieving good or excellent distension according to mannitol oral contrast volume ingested

|  | **Volume less or equal than 1L**  **N* (%)** | **Volume more than 1L**  **N* (%)** | **Difference****  **% (95% CI)** |
| --- | --- | --- | --- |
| Patients | 17/33 (52) | 18/32 (56) | -5 (-29 to 19) |
| Segments |  |  |  |
| Duodenum | 5/33 (15) | 5/32 (16) |  |
| Jejunum | 11/33 (33) | 15/32 (47) | -14 (-37 to 10) |
| Ileum | 24/33 (73) | 25/32 (78) |  |
| Terminal_ileum | 17/33 (52) | 19/32 (59) |  |
| Caecum | 8/19 (42) | 12/30 (40) |  |
| Ascending_C | 18/27 (67) | 23/31 (74) |  |
| Transverse_C | 17/29 (59) | 23/32 (72) | -13 (-37 to 10) |

*In some patients segments had been excised and so could not be assessed.

** Volume less or equal than 1L vs Volume more than 1L (The statistical power is limited and therefore unable to show a statistical difference between proportions)

## Table 3: Excellent or good distension overall and by segment for the two readers

|  |  | **Reader 1** | **Reader 2** | **Agreement** | **Gwet's AC** |
| --- | --- | --- | --- | --- | --- |
| **Segment** | **N*** | **N (%)** | **N (%)** | **% (95% CI)** |  |
| Overall | 105 | 48 (45) | 45 (42) | 82 (75 to 90) | 0.65 |
| Duodenum | 105 | 11 (10) | 9 (8) | 87 (80 to 93) | 0.84 |
| Jejunum | 105 | 21 (20) | 22 (21) | 79 (71 to 86) | 0.68 |
| Ileum | 105 | 63 (59) | 70 (65) | 73 (64 to 81) | 0.49 |
| Terminal_ileum | 105 | 52 (49) | 30 (28) | 68 (59 to 77) | 0.40 |
| Caecum | 80 | 30 (37) | 31 (39) | 84 (75 to 92) | 0.69 |
| Ascending_C | 94 | 61 (64) | 53 (55) | 77 (69 to 86) | 0.56 |
| Transverse_C | 101 | 53 (51) | 33 (32) | 69 (60 to 78) | 0.40 |

*In some patients segments had been excised and so could not be assessed.

## Table 4:

## Patient symptoms by contrast agent and time point following MRE

|  | **Mannitol (N=85)** | | | **Klean Prep (N=45)** | | |
| --- | --- | --- | --- | --- | --- | --- |
|  | **Very tolerable** | **Moderately tolerable** | **Not tolerable** | **Very tolerable** | **Moderately tolerable** | **Not tolerable** |
|  | **n/N^a^ (%)** | **n/N^a^ (%)** | **n/N^a^ (%)** | **n/N^a^ (%)** | **n/N^a^ (%)** | **n/N^a^ (%)** |
| Symptoms immediately following MRE | | | | | | |
| A feeling of fullness | 37/84 (44*) | 46/84 (55) | 1/84 (1) | 12/45 (27*) | 32/45 (71) | 1/45 (2) |
| Regurgitation | 62/83 (75) | 18/83 (22) | 3/83 (4) | 31/43 (72) | 8/43 (19) | 4/43 (9) |
| Vomiting | 72/81 (89) | 7/81 (9) | 2/81 (2) | 38/44 (86) | 3/44 (7) | 3/44 (7) |
| Abdominal pain/ spasms | 50/82 (61) | 28/82 (34) | 4/82 (5) | 25/43 (58) | 14/43 (33) | 4/43 (9) |
| Diarrhoea | 54/82 (66) | 24/82 (29) | 4/82 (5) | 25/44 (57) | 12/44 (27) | 7/44 (16) |
| Symptoms for the 2 days following MRE | | | | | | |
| Flatulence | 23/48 (48) | 20/48 (42) | 5/48 (10) | 22/42 (52) | 15/42 (36) | 5/42 (12) |
| Regurgitation | 39/43 (91) | 4/43 (9) | 0/43 (0) | 33/41 (80) | 6/41 (15) | 2/41 (5) |
| Vomiting | 39/44 (89) | 3/44 (7) | 2/44 (5) | 36/41 (88) | 3/41 (7) | 2/41 (5) |
| Abdominal pain/ spasms | 15/49 (31) | 29/49 (59) | 5/49 (10) | 18/42 (43) | 19/42 (45) | 5/42 (12) |
| Diarrhoea | 24/55 (44) | 22/55 (40) | 9/55 (16) | 14/44 (32) | 24/44 (55) | 6/44 (14) |

a The number of patients answering each survey question varies, so numbers and percentages are given for those responding.

*The only significant difference in experience between patients receiving mannitol and those receiving Klean Prep was in the feeling of fullness immediately after contrast, where 17% more patients (95% CI 0.6 to 34%) had a very tolerable feeling of fullness with mannitol compared to Klean Prep.

## Table 5: Patient experience according to mannitol oral contrast volume ingested

|  | **Volume less or equal than 1L**  **(N=40)** | | | **Volume more than 1L**  **(N=45)** | | |
| --- | --- | --- | --- | --- | --- | --- |
|  | **Very tolerable** | **Moderately tolerable** | **Not tolerable** | **Very tolerable** | **Moderately tolerable** | **Not tolerable** |
|  | **n/N^a^ (%)** | **n/N^a^ (%)** | **n/N^a^ (%)** | **n/N^a^ (%)** | **n/N^a^ (%)** | **n/N^a^ (%)** |
| Symptoms immediately following MRE | | | | | | |
| A feeling of fullness | 18/39 (46) | 20/39 (51) | 1/39 (3) | 19/45 (42) | 26/45 (58) | 0/45 (0) |
| Regurgitation | 30/40 (75) | 10/40 (25) | 0/40 (0) | 32/43 (74) | 8/43 (19) | 3/43 (7) |
| Vomiting | 34/38 (89) | 4/38 (11) | 0/38 (0) | 38/43 (88) | 3/43 (7) | 2/43 (5) |
| Abdominal pain/ spasms | 25/39 (64) | 13/39 (33) | 1/39 (3) | 25/43 (58) | 15/43 (35) | 3/43 (7) |
| Diarrhoea | 23/39 (59) | 14/39 (36) | 2/39 (5) | 31/43 (72) | 10/43 (23) | 2/43 (5) |
| Symptoms up to 2 days following MRE | | | | | | |
| Flatulence | 14/26 (54) | 12/26 (46) | 0/26 (0) | 9/22 (41) | 8/22 (36) | 5/22 (23) |
| Regurgitation | 23/24 (96) | 1/24 (4) | 0/24 (0) | 16/19 (84) | 3/19 (16) | 0/19 (0) |
| Vomiting | 23/25 (92) | 2/25 (8) | 0/25 (0) | 16/19 (84) | 1/19 (5) | 2/19 (11) |
| Abdominal pain/ spasms | 9/26 (35) | 14/26 (54) | 3/26 (12) | 6/23 (26) | 15/23 (65) | 2/23 (9) |
| Diarrhoea | 14/30 (47) | 11/30 (37) | 5/30 (17) | 10/25 (40) | 11/25 (44) | 4/25 (16) |

The number of patients answering each survey question varies, so numbers and percentages are given for those responding.

## Figure 1: Comparison of patient symptoms dependent on oral preparation ((a)mannitol based and (b) PEG based) immediately after and up to two days after the MRE
