# Supplementary material for: Influence of oral contrast type and volume on patient experience and quality of luminal distension at MR Enterography in Crohn’s disease: an observational study of patients recruited to the METRIC trial
Source: Eur Radiol. 2022 Mar 3;32(8):5075–85. doi: 10.1007/s00330-022-08614-9 (PMC9279188; doi:10.1007/s00330-022-08614-9)
Supplement: Supplementary file 2 — (PDF 125 kb) [file 330_2022_8614_MOESM2_ESM.pdf]

European Radiology

Influence of oral contrast type and volume on patient experience and quality of luminal distension at MR Enterography in Crohn’s disease: An observational study of patients recruited to the *BLINDED* trial

--Manuscript Draft--

**Manuscript Number:**	EURA-D-21-04028R1
**Full Title:**	Influence of oral contrast type and volume on patient experience and quality of luminal distension at MR Enterography in Crohn’s disease: An observational study of patients recruited to the *BLINDED* trial
**Article Type:**	Original Article
**Funding Information:**	Health Technology Assessment Programme (11/23/01) — Prof. Stuart Taylor
**Abstract:**	Objectives<br>To compare the distention quality and patient experience of oral mannitol and polyethylene glycol (PEG) for MRE.<br>Methods<br>This study is a retrospective, observational study of a subset of patients enrolled in a multicenter, prospective trial evaluating the diagnostic accuracy of MRE for small bowel Crohn's. Overall and segmental small bowel distention, from 105 patients (64F, mean age 37) was scored from 0=poor to 4=excellent by two experienced observers (68[65%]mannitol and 37[35%]PEG). Additionally, 130 patients (77F, mean age 34) completed a questionnaire rating tolerability of various symptoms immediately and 2 days after MRE (85[65%]receiving mannitol 45[35%]receiving PEG). Distension was compared between agents, and between those ingesting ≤1L or >1L of mannitol using the test of proportions. Tolerability grades were collapsed into “Very tolerable”, “Moderately tolerable” and “Not tolerable”<br>Results<br>Per patient distension quality was similar between agents (“excellent” or “good” in 54%[37/68] verses 46%[17/37] with mannitol and PEG respectively. Jejunal distension was significantly better with mannitol compared to PEG (40% [27/68] verses 14%[5/37] rated as excellent or good respectively). There was no significant difference according to the volume of mannitol ingested.<br>Symptom tolerability was comparable between agents, although fullness following MRE was graded as ‘very tolerable’ in 27%[12/45] of patients ingesting PEG, verses 44%[37/84] ingesting mannitol, difference 17%(95%CI 0.6 to 34%).<br>Conclusion<br>Mannitol-based solutions and PEG generally achieve comparable distension quality and side effect profiles, although jejunal distension is better quality with mannitol. Neither distension quality nor side-effect profile are altered by ingestion of more than 1L of mannitol.
**Corresponding Author:**	Stuart A Taylor<br>University College London<br>London, UNITED KINGDOM
**Corresponding Author Secondary Information:**	
**Corresponding Author's Institution:**	University College London
**Corresponding Author's Secondary Institution:**	
**First Author:**	Gauraang Bhatnagar, FRCR
**First Author Secondary Information:**	
**Order of Authors:**	Gauraang Bhatnagar, FRCR
	Sue Mallett

Powered by Editorial Manager® and ProduXion Manager® from Aries Systems Corporation

	Laura Quinn, MSc
	Rajapandian Ilangovan, FRCR
	Uday Patel, PhD
	Asif Jaffer, FRCR
	Christopher Pawley, FRCR
	Arun Gupta, FRCR
	Anthony Higginson, FRCR
	Andrew Slater, FRCR
	Damian Tolan, FRCR
	Ian Zealley, FRCR
	Steve Halligan, FMedSci
	Stuart Taylor, FRCR
**Order of Authors Secondary Information:**	
**Author Comments:**	Dear Editor,<br><br>Please see attached a revised resubmission of our manuscript entitled “Influence of oral contrast type and volume on patient experience and quality of luminal distension at MR Enterography in Crohn’s disease: An observational study of patients recruited to the BLINDED trial”. We have read the comments raised during the second review of this paper by your editors and have made all suggested positive changes to the manuscript. We include a detailed reply to the review comments as part of the submission. We hope the changes have satisfactorily modified the manuscript for your readership. We look forward to your re-review.<br><br>Yours sincerely,<br><br>Dr. Gauraang Bhatnagar<br>Professor Stuart Taylor

Powered by Editorial Manager® and ProduXion Manager® from Aries Systems Corporation

Reviewer #1: European Radiology
EURA-D-21-04028
Influence of oral contrast type and volume on patient experience and quality of luminal distension at MR Enterography in Crohn's disease: An observational study of patients recruited to the "Blinded" trial.

	Reviewer comment	Author response
REV 1.1	The opening sentence of the introduction is debatable. There are no data that support that MRE is the most sensitive and specific cross-sectional imaging modality for diagnosing and staging of small bowel Crohn's disease. I and many others would argue that CTE is just as sensitive and specific. Yes, there are some data that suggest that MRE can determine the fibrotic component of a stricture, but this has not been universally replicated. MRE in many instances is preferable over CTE as there is no ionizing radiation. There is however a very legitimate role of CTE for Crohn's disease, especially in the acute situation or where MR is not readily available. Lastly, an MRE with excessive patient motion and peristalsis, NOT an uncommon occurrence, is useless. That almost never happens with CTE. Please modify this statement	Amended as suggested.
REV 1.2	Please reorder the methods section in the abstract. The study is a retrospective, observational study of a subset of patients enrolled in a multicenter, prospective study evaluating the diagnostic accuracy of MRE for small bowel Crohn's disease. Just starting with "Overall and segmental MRE small bowel distension…" isn't the best.	Amended as suggested.
REV 1.3	One thing that did not occur to me when I first reviewed the manuscript was whether there was any assessment of bowel distension as the MR sequences were performed. My impression is that the first sequences, in our case the initial coronal HASTE sequences, the mid to distal ileum is most distended when compared to the last pulse sequence in the exam, the 7 minute delayed post contrast VIBE sequence. It would have been interesting to see if there were any differences between the first and last sequence. This was not the design of the study and I'm not asking the authors to redo their assessment (as an author myself, I despise a reviewer asking me to do a different study than the one I did). But, it might be interesting for the authors to look at the sequences, from first to last, and	Thank you for this comment. We agree this will be useful additional work and have added a comment to the discussion.

	see if one enteric agent more consistently distended the bowel. This could be a separate, new investigation worthy of publication.	
REV 1.4	While not necessary to the discussion, the results are likely applicable to CTE and could be added to the discussion.	Amended as suggested.

Title Page (Title, Authors, Institutions, Contact Information)

Influence of oral contrast type and volume on patient experience and quality of luminal distension at MR Enterography in Crohn's disease: An observational study of patients recruited to the METRIC trial

Authors

Gauraang **Bhatnagar**[1] FRCR, Sue **Mallett**[1] D.Phil, Laura **Quinn**[2] MSc, Rajapandian **Ilangovan**[3] FRCR, Uday **Patel**[3] PhD, Asif **Jaffer**[1] FRCR, Christopher **Pawley**[1] FRCR, Arun **Gupta**[3] FRCR, Anthony **Higginson**[4] FRCR, Andrew **Slater**[5] FRCR, Damian **Tolan**[6] FRCR, Ian **Zealley**[7] FRCR, Steve **Halligan**[1] FMedSci, Stuart **Taylor**[1] FRCR on behalf of the METRIC study investigators

Author affiliations

1. Centre for Medical Imaging, University College London, Charles Bell House, 43-45 Foley Street, W1W 7TS
2. Institute of Applied Health Research, NIHR Birmingham Biomedical Research Centre, College of Medical and Dental Sciences, University of Birmingham, Edgbaston, Birmingham, B15 2TT, UK
3. Intestinal Imaging Centre, St Mark's Hospital, LNWUH NHS Trust, Harrow, UK
4. Department of Radiology, Portsmouth Hospitals NHS Trust, Portsmouth, UK
5. Department of Radiology, Oxford University Hospitals NHS Trust, Oxford, UK
6. Department of Radiology, St James's University Hospital, Leeds Teaching Hospitals NHS Trust, Beckett Street, Leeds, LS9 7TF, UK
7. Department of Radiology, Ninewells Hospital, Dundee, DD1 9SY, Scotland, UK

Corresponding author

Stuart A Taylor, Centre for Medical Imaging, University College London, Charles Bell House, 43-45 Foley Street, W1W 7TS. Email stuart.taylor@ucl.ac.uk. Tel 07960169321

Influence of oral contrast type and volume on patient experience and quality of luminal distension at MR Enterography in Crohn's disease: An observational study of patients recruited to the METRIC trial

Abstract

Objectives

To compare the distention quality and patient experience of oral mannitol and polyethylene glycol (PEG) for MRE.

Methods

This study is a retrospective, observational study of a subset of patients enrolled in a multicenter, prospective trial evaluating the diagnostic accuracy of MRE for small bowel Crohn's. Overall and segmental MRE small bowel distention, from 105 patients (64F, mean age 37) was scored from 0=poor to 4=excellent by two experienced observers (68[65%] mannitol and 37[35%] PEG). Additionally, 130 patients (77F, mean age 34) completed a questionnaire rating tolerability of various symptoms immediately and 2 days after MRE (85[65%] receiving mannitol 45[35%] receiving PEG). Distension was compared between agents, and between those ingesting ≤1L or >1L of mannitol using the test of proportions. Tolerability grades were collapsed into "Very tolerable", "Moderately tolerable" and "Not tolerable"

Results

Per patient distension quality was similar between agents ("excellent" or "good" in 54%[37/68] verses 46%[17/37] with mannitol and PEG respectively. Jejunal distension was significantly better with mannitol compared to PEG (40%

[27/68] verses 14%[5/37] rated as excellent or good respectively). There was no significant difference according to the volume of mannitol ingested.

Symptom tolerability was comparable between agents, although fullness following MRE was graded as 'very tolerable' in 27%[12/45] of patients ingesting PEG, verses 44%[37/84] ingesting mannitol, difference 17%(95%CI 0.6 to 34%).

Conclusion

Mannitol-based solutions and PEG generally achieve comparable distension quality and side effect profiles, although jejunal distension is better quality with mannitol. Neither distension quality nor side-effect profile are altered by ingestion of more than 1L of mannitol.

Keywords

1. Crohn's Disease
2. Diagnostic imaging
3. Magnetic Resonance Imaging

Key points

- Mannitol-based and PEG based oral preparation agents generally achieve comparable distension quality for MRE with the exception of the jejunum which is better distended with mannitol.
- Mannitol-based and PEG based oral preparation agents used for MRE have similar side effect profiles.

- Neither distension quality nor side-effect profile are altered by ingestion of more than 1L of mannitol.

Abbreviations

CD - Crohn’s disease

ESGAR - European Society of Gastrointestinal and Abdominal Radiology

METRIC Trial – MR Enterography or ulTRasound In Crohn's disease
MRE - Magnetic Resonance Enterography

PEG - Polyethylene Glycol

SBU - Small bowel ultrasound

Introduction

Cross sectional imaging is sensitive and specific for diagnosing and staging of small bowel Crohn's disease (CD). Magnetic resonance enterography (MRE) has the advantage of not exposing patients to ionising radiation. [1]. It relies upon the combination of good small bowel distension and multi-parametric sequences to accurately identify disease and phenotype as either predominantly inflammatory or fibrostenotic [2, 3]. Diagnostic accuracy pivots on the quality of luminal distension; poor distension can both conceal or mimic disease, leading to misdiagnosis. Distension is influenced by the type and volume of oral preparation agent ingested and a variety of protocols are used clinically [4–7]. A recent literature review by the European Society of Gastrointestinal and Abdominal Radiology (ESGAR) found no evidence for superiority of one oral preparation over another, and made no specific recommendation on either the optimal agent, nor ingested volume [8].

Whilst MRE is generally well tolerated, compared to small bowel ultrasound (SBU), it is more burdensome and causes symptoms, such that SBU is usually preferred by patients [9]. Gastrointestinal effects related to the agent are most commonly cited by patients as the least acceptable characteristic of MRE [9].

The existing literature investigating oral preparation agents has largely focused on a small number of healthy individuals at single centres [5, 10–15]. Findings may not generalise to the (often) symptomatic patients undergoing MRE. Indeed,

surprisingly few studies have investigated oral contrast agents in patients [16–18].

We conducted a prospective multi-centre study comparing the diagnostic accuracy of MRE with SBU in Crohn's disease [1, 19] . The study afforded the pragmatic opportunity to prospectively compare two commonly used MRE oral contrast agents, mannitol and polyethylene glycol (PEG) exactly as they are employed in clinical practice, and was a pre-specified secondary outcome [19, 20]. Specifically, the aims of the current study were to compare, 1) distension quality and, 2) patient symptoms, according to the agent ingested. We also investigated the influence of ingested volume on image distention quality and patients' symptoms.

Materials and methods

Study Population

This study was conducted as a pre-specified sub-study of a larger multi-centre, prospective cohort trial investigating the sensitivity of MRE and SBU. The trial recruited two patient cohorts: (1) newly diagnosed and, (2) established disease, clinically suspected of luminal relapse [1, 19]. Full ethical permission was obtained (NRES Committee September 2013 reference 13/SC/0394).

The current study was a sample of convenience based on, 1) receipt of MRE datasets from recruitment sites for central distension scoring during the course of the trial up until October 2015, 2) available information on oral contrast agent

type and volume ingested and, 3) return of completed patient experience questionnaires.

The study cohort consisted of 114 (34%) of the 335 patients recruited to the main diagnostic accuracy trial. Of these 114, 9 patients were excluded subsequently (6 did not have a diagnosis of CD and 3 withdrew from the trial). The final cohort consisted of 105 patients recruited across 6 sites. Overall, 68 [65%] received mannitol based oral preparation and 37 [35%] received PEG based oral preparation. A subset of 66 patients from the current study has been reported in part previously [20].

Study Design

Imaging Protocol

The main diagnostic accuracy trial was a pragmatic trial. As such, all recruitment sites used their usual clinical protocol for all MRE examinations. There was no specific stipulation as to the type of oral preparation agent to be used or volume to be ingested.

This study included patients recruited from six of the eight centres that took part in the main trial. The remaining two centres had not provided data by the submission deadline for this substudy due to delays in commencing recruitment. Four of the six sites used a mannitol based oral preparation regimen (two sites used mannitol 2.5% alone, one site used mannitol 2.5% & 0.2% Locust Bean Gum and one site used mannitol 2.5% & 2 scoops Carobel, Cow & Gate, Nutricia Ltd.,). Two sites utilised a PEG oral preparation without additives (69g Klean prep/litre, Helsinn-Birex pharmaceuticals Ltd.). Full details of the differing oral

prep regimens employed at all sites is provided in Appendix 8. Patients were instructed to drink the provided volume of oral preparation (1.5-2L) at a steady rate over a 45-60 minute period according to tolerance, and encouraged by radiography staff at regular intervals.

A minimum dataset of sequences was acquired including T2-weighted images with and without fat saturation, steady state free precession gradient echo images, diffusion weighted images and T1-weighted images after intravenous gadolinium injection (see Appendix 1 for minimal MRE dataset).

Recording MRE oral preparation details

Recruitment sites were requested to record prospectively the exact volume of ingested contrast agent.

Patient experience questionnaires

Patients recruited to the main diagnostic accuracy trial were invited to complete a three-part questionnaire asking their experience of various symptoms before and after oral preparation. The questionnaire was given to participants by radiographers. Patients were asked to complete a baseline questionnaire the day of their MRE but before ingesting the oral contrast, and to then complete a second questionnaire immediately after MRE. These were then handed to radiography staff. The third questionnaire was completed two days later, to capture symptoms for 48 hours post MRE. Patients were asked to return this either by hand or mail (stamped, addressed envelopes were provided). At each of the three time points, participants were asked to rate tolerability (“not at all tolerable”, “somewhat tolerable”, “moderately tolerable”, “very tolerable”) and

record symptoms of fullness, regurgitation, vomiting, abdominal pains/spasms and diarrhoea.

The questionnaire is reproduced in Appendix 2.

Grading of Bowel distension

All MRE examinations were anonymised (for patient and site specific information) and uploaded to an online viewing platform (Biotronics 3Dnet, Biotronics 3D)

Two Consultant gastrointestinal radiologists reporting MRE as part of their routine clinical work at a tertiary referral centre for 10 and 4 years respectively, reviewed all MREs independent of each other and performed qualitative distension grading in isolation. Observers were blinded to the oral contrast agent used and its volume.

The small bowel was divided into the duodenum, jejunum, ileum, and terminal ileum). The terminal ileum was defined as the terminal 10cm of small bowel. The jejunum was defined as the proximal bowel lying largely to the left of a diagonal line drawn from the right lower quadrant to the left lower quadrant, demonstrating a typical "feathery" fold pattern, and the ileum as the bowel interposed between jejunum and terminal ileum[19]. Right colonic segments (caecum, ascending colon and transverse colon) were defined as described previously [21].

Segmental distension was graded qualitatively using the methods described by Saini et al [22] . Each small bowel and right colonic segment (caecum, ascending colon, transverse colon), was graded independently by each observer on a 5 point scale: 0 - very poor distension; 1 - poor distension; 2 - fair distension; 3 - good distension; and 4 - excellent distension [5, 7, 15, 22]. The reviewers were instructed to use the entire image set as part of their assessment..

Observers also scored the overall per-patient quality of small bowel distension using the same scale. Observers were free to use all MRE sequences when making their grading decision.

Statistical analysis

The frequency of “excellent” or “good” distension according to the type of oral contrast was calculated on a per-patient level and for individual intestinal segments. In cases of reader disagreement, the best distension score of the two was used for the main analysis but results from each individual reader are also presented . Per-patient and segmental distension scores were compared between the two oral contrast groups using the test of proportions. Distension scores were also compared according to the volume of mannitol ingested (1L or less vs. more than 1L). The distribution of data for the volume of PEG ingested was insufficient to undertake meaningful analysis for this agent.

For the purposes of analysis, tolerability grades were collapsed as follows; (1) Very tolerable ("I did not experience this symptom" and "very tolerable"), (2) Moderately tolerable (combining "moderately" and "somewhat" tolerable) and (3) Not tolerable ("not at all tolerable") and compared according to oral contrast agent and volume of mannitol ingested (1L or less vs. more than 1L).

Inter-observer variability for distension scores were analysed using Gwet's chance-adjusted agreement coefficient [23]. Strength of agreement was interpreted using the Landis and Koch criteria [24]: < 0.00 = poor, 0.00–0.20 = slight, 0.21–0.40 = fair, 0.41–0.60 = moderate, 0.61–0.80 = substantial, and 0.81–1.00 = excellent.

Results

Distension cohort

Patient characteristics

Detailed patient characteristics are provided in Appendix 3. Importantly there was no important differences between the groups in potential confounders which could influence distension, notably presence of stenosis, prior resection and disease activity (as measured by the HBI and CRP).

The volumes of mannitol and PEG based oral preparation ingested is presented in Appendix 4. Of the 68 patients who ingested mannitol, 3 did not have information on volume ingested (and so were excluded from volume analysis). Overall, patients ingested between 200ml and 1.8L, and 49% [32/65] ingested more than 1L. Of the 37 patients who ingested PEG, the volume ranged between 300ml to 1.5L and 11% [4/35] patients drank more than 1L. Overall, 34% [22/65] ingested exactly 1L of mannitol and 35% [13/37] ingested exactly 1L of PEG.

Qualitative distension assessment

Quality of luminal distension according to mannitol or PEG oral preparation

Using the best distension score of the two readers, there was no significant difference in the proportion of patients achieving excellent or good distention between mannitol or PEG based preparations. Specifically, per-patient distension with mannitol based preparation was rated as excellent or good in 54% [37/68] verses 46% [17/37] with PEG based preparation (Percentage difference [95%CI] 8 [-11to 28]) (Table 1).

At a segmental level, ileal distension quality was greater for both preparations, followed by the terminal ileum and then jejunum (Table 1). Jejunal and transverse colon segmental distension were significantly better distended in the mannitol group in comparison to the PEG group (Table 1). This pattern was consistent for both individual readers, reaching statistical significance for reader 2 in the jejunum (95% CI of difference in distension 11% to 37%) and reader 1 in the transverse colon (95% CI of difference in distension 5% to 44%) (Appendix 9).

In general, the distension quality grading were comparable across both individual readers with the exception of the TI (appendix 9). Reader 1 graded a greater proportion of TI segments as good/excellent distension on both preparations (Mannitol 36 (53%) and PEG 15 (41%) versus Reader 2 (Mannitol 20 (29%) and PEG (24%)).

Overall Inter-observer variability

Table 3 demonstrates inter-reader agreement for patients/segments where the two readers rated distention as “excellent” or “good”. On a per-patient basis, there was substantial agreement between readers, with reader 1 rating 45% [48/105] and reader 2 rating 42% [45/102] of MREs as achieving “excellent” or “good” distention. There was excellent inter-reader agreement in assessing duodenal distension (Gwet's AC=0.84 Gwet’s AC 0.84 (10% and 8% assessed good/excellent distension by the two readers)) but only fair for terminal ileal distension (Gwet's AC=0.40 (49% and 28% assessed good/excellent distension by

the two readers)). There was substantial agreement in assessing jejunal distension (Gwet's AC = 0.68 assessed as good to excellent distension in only 20 and 21% by the two readers). There was moderate agreement for the ileum (Gwet's AC = 0.49 (59% and 65% assessed good/excellent distension by the two readers)).

Impact of oral Mannitol volume ingested

There was no significant difference in distension quality either overall or on a segmental basis according to the volume of mannitol ingested (1L or less vs more than 1L) (Table 2). Ileal distension quality was most frequently rated the greatest for both cohorts. The Jejunum achieved good/adequate distension in 15/32 (47%) ingesting more than 1L of mannitol versus 11/33 (33%) of patients ingesting 1L or less but this difference was not of statistical significance (-14% (-37 to 10)). Of note, the sample size was limited and therefore a test of proportions was not appropriate.

Patient symptoms

Overall, 143 (43%) of the 335 patients recruited to the main diagnostic accuracy trial had information on oral contrast type and volume ingested as well as providing a completed questionnaire immediately after MRE. Of these, 13 patients were excluded (10 patients did not have a diagnosis of CD and 3 withdrew from the trial). The final cohort consisted of 130 patients recruited across 6 sites, with 85[65%] receiving mannitol based oral preparation and 45[35%] receiving PEG based oral preparation. Full patient characteristics are

provided in Appendix 5. 78 (60%) of these 130 patients were also part of the cohort included in the qualitative distension study.

The delayed patient symptom questionnaire (for symptoms over the two-days following MRE) had a variable return rate: mannitol 44/85 [52%] and PEG 44/45 [98%]. All patients recording any symptom as 'not tolerable' immediately after MRE completed the delayed symptom questionnaire .

The volumes of mannitol and PEG based oral preparation ingested is shown in Appendix 6. The volume of mannitol ingested ranged between 200ml and 1.8L with 34% [29/85] drinking 1L. The volume PEG ingested ranged between 300ml to 1.5L, with 33% [15/45] drinking 1L.

Patient symptoms according to mannitol or PEG oral preparation

Baseline symptoms were comparable between mannitol and PEG groups (Appendix 7).

Tolerability of symptoms immediately after MRE and over the next 2 days is shown in Table 4 and graphically in Figure 1.

In general, symptom tolerability immediately after the MRE were comparable between the two preparations. For the mannitol group, vomiting and regurgitation were the best tolerated symptoms and abdominal pain the least. Symptoms of fullness were graded 'very tolerable' in just 27% [12/45]) of patients ingesting PEG, a significantly lower proportion than for mannitol (44% [37/84]) , a 17% difference (95%CI 0.6-34%))

For both preparations, tolerability of abdominal pain and diarrhoea was generally rated worse after 2 days then immediately after MRE, and again largely comparable between preparations. For example, 2 days post MRE, patients reported abdominal pain as very tolerable in 31% (15/49) and 43% [18/42] after ingesting mannitol and PEG respectively, a decrease from 61% (50/82) and 58% (25/43) immediately after MRE. Similarly, diarrhoea was worse after 2 days for both preparations, for example 32% (14/44) reporting it as very tolerable 2 days after PEG ingestion compared to 57% (25/44) immediately after MRE. Regurgitation improved after 2 days.

Patient symptoms depending volume of oral preparation ingested

The influence of ingested mannitol volume on patient symptoms is shown in Table 5. The response rate for the delayed symptom questionnaire was 30/40[75%] and 25/45[56%] for the "1L or less" and "more than 1L" groups respectively.

In general, symptoms immediately after MRE were comparable between those ingesting 1L or less compared to those ingesting more than 1 L.

Diarrhoea immediately after MRE was rated 'very tolerable' by 59% [23/39] of patient drinking 1L or less and 72% [31/43] of patients drinking more than 1L; not statistically significant.

Similarly, symptoms up to two days after MRE were comparable between the two volumes ingested. For example, abdominal pain/spasms were 'very tolerable' in 35% [9/26] of patients drinking 1L or less and 26% [6/23] of

patients drinking more than 1L. Diarrhoea, was ‘very tolerable’ in 47% [14/30] of patient drinking 1L or less and 40% [10/25] of patients drinking more than 1L

The same pattern of worsening tolerability of abdominal pain and diarrhoea but improved regurgitation after 2 days was observed in both volume groups.

Discussion

We conducted a large multi-centre, prospective diagnostic accuracy trial investigating the sensitivity of MRE and SBU. This afforded the opportunity to prospectively assess the quality of bowel distension achieved in representative clinical practice by two of the most commonly used MRE distention agents, and to compare symptoms following ingestion. The results may also translate to other luminal investigations requiring luminal distension such as CT enterography and Hydrosonography.

To date, the majority of previous literature pertaining to oral contrast agents has reported healthy volunteers who are unlikely to represent patients commonly undergoing MRE [5, 10–15], or limited to retrospective studies of small numbers at single centres [22, 25, 26]. In this regard, our work adds to the current literature.

We found that, overall, there were no major differences in distention quality between either mannitol based preparations or PEG. However, we did find some evidence that whilst jejunal distension remains challenging, it is more commonly good or excellent quality with mannitol (40%) compared to PEG (14%). This is potentially an important observation given difficulties with jejunal distention during MRE (as opposed to MR enteroclysis) and the potential impact on diagnostic accuracy. Importantly, the two groups were generally well matched in terms of presentation (new diagnosis verses relapse), presence of stenosis, and history of prior surgical resection, which increases our confidence that our findings are real and not secondary to unequal disease phenotypes across

cohorts. Although the colon is not the primary target for MRE, it is interesting to note superior transverse colonic distension with mannitol.

We also found ingesting more than 1L of mannitol did not confer any beneficial effect. This concurs with Ajaj et al who reported that in a study of 10 volunteers, 1000, 1200, and 1500 ml of mannitol all gave similar quality distension[5]. Overall, our data suggest there is no need for patients to ingest more than 1l of oral contrast. Perhaps surprisingly, we did not find any difference in the immediate or delayed symptoms experienced by patients, regardless of the volume of contrast. There was perhaps a trend for greater diarrhoea in those drinking more contrast, but this was not statistically significant perhaps due to underpowering.

We also found mannitol and PEG were similarly tolerable, although patients ingesting PEG reported that fullness was significantly less tolerable immediately after MRE compared to those ingesting mannitol. We note that the proportion of patients ingesting 1l or more was lower in the PEG cohort then the mannitol cohort. Whilst the exact reasons for this observation are uncertain, it is possible the greater feelings of fullness in the PEG group led to reduced overall intake.

Of note, abdominal pain and diarrhoea increased over the 2 days after MRE compared to immediately afterwards. This is perhaps unsurprising as it takes time for contrast to traverse the gut, and concurs with a recent study in which oral contrast was rated the most unpleasant component of MRE; 18% of patients

take longer than 1 day to recover [9]. Patients should be warned of this prior to MRE.

There are no published studies comparing mannitol and PEG in MRE but a recently published randomized controlled trial did compare the two preparations in 70 patients undergoing CT enterography at a single-centre. Each patient underwent 2L of PEG bowel preparation prior to ingesting either 1.5L of mannitol or PEG solution. The study reported no significant differences in the quality of luminal distension between the agents but stated that patients undergoing mannitol preparation reported nausea as more tolerable, the taste as more acceptable, and were more willing to ingest again compared to patients undergoing PEG preparation. [27]

Our study has limitations. We investigated the impact of mannitol and PEG based oral preparations alone, as these were the two agents utilised at centres recruiting to the main diagnostic accuracy trial. The number of patients undergoing PEG based oral preparation were smaller, and as a result we were unable to incorporate the PEG cohort in assessment of the impact of ingested oral volume on either luminal distension or patient experience. Furthermore, while we had a good proportion of delayed patient experience questionnaires returned in the PEG cohort, this was much reduced in the mannitol cohort, which impairs comparison for delayed symptoms. This may in part reflect different tenacity of individual recruitment sites when encouraging patients to return questionnaires but risks some bias. Whilst we compare the main agents of the oral preparation, we acknowledge that some mannitol preparations utilised

additives such as LBG or Carobel in small quantities and we have not assessed the specific contribution of these additives. Both readers for the qualitative assessment of luminal distension work at one centre which employed a Mannitol based preparation. This may introduce some bias related to their prior experience. For practical reasons other centres did not provide readers for this substudy although that would have been optimal. Agreement between readers was generally good, although less so for the terminal ileum in particular. Reassuringly though both readers were consistent in the relative grading of distension quality between the two preparations for all segments so any disagreement in absolute levels of distension did not impact on our main conclusion. Whilst the main trial evaluated the diagnostic accuracy of MRE (and SBU), this substudy was underpowered to draw conclusions on whether the differing quality of luminal distension affected the overall diagnostic accuracy. This would be a useful topic for further research. However it is reassuring that although distension quality was judged as excellent in slightly over 50% of terminal ileal segments, the results on the main trail showed MRE has high sensitivity for terminal ileal Crohn's disease, suggesting accurate diagnosis does not always require optimal distension. Anecdotally, the segmental small bowel distension will alter throughout the MRI acquisition (as the study typically takes 30-45 minutes to acquire), it would be of interest to review whether this change in segmental distension is different for differing luminal preparation agents; this was felt to be outside the remit of this study.

Patients who reported at least one symptom as 'not tolerable' generally completed the day 2 questionnaire, whereas those less affected completed fewer.

This may introduce spectrum bias, with a greater proportion returning this questionnaire more likely to experience less tolerable symptoms. Ours was a convenience sample based on data return, which could induce bias. Reassuringly, we found no major difference between PEG and mannitol cohorts regarding disease phenotype or baseline symptom level. Although to our knowledge ours is the largest prospective patient study on this topic to date, we did not perform a prior power calculation and so some of our comparisons are likely unpowered, for example the effects of agent volume on distension and symptoms.

In summary, mannitol-based solutions and PEG generally achieve comparable distension quality and side effect profiles, although jejunal distension is more frequently of better quality with mannitol. Distension quality is not improved by ingestion of more than 1l, although doing so does not adversely influence patent tolerability.

Funding
This study has received funding by NIHR HTA 11/23/01 and the UCLH NIHR BRC.

Guarantor:
The scientific guarantor of this publication is Professor Stuart A Taylor.

Conflict of Interest:
The authors of this manuscript declare no relationships with any companies whose products or services may be related to the subject matter of the article.

Statistics and Biometry:
Professor Sue Mallett kindly provided statistical advice for this manuscript.

Informed Consent:
Written informed consent was obtained from all subjects (patients) in this study.

Ethical Approval:
Institutional Review Board approval was obtained.

Study subjects or cohorts overlap:
A subset of 66 patients from this study have been previously reported in Taylor SA, Mallett S, Bhatnagar G, et al. (2019) Magnetic resonance enterography compared with ultrasonography in newly diagnosed and relapsing crohn's disease patients: The METRIC diagnostic accuracy study. Health Technol Assess (Rockv) 23:vii–161. doi: 10.3310/hta23420.

Methodology

- prospective
- observational
- multicenter study

References

1. Taylor SA, Mallett S, Bhatnagar G, et al. (2018) Diagnostic accuracy of magnetic resonance enterography and small bowel ultrasound for the extent and activity of newly diagnosed and relapsed Crohn ' s disease (METRIC): a multicentre trial. Lancet Gastroenterol Hepatol 3:548–58. doi: 10.1016/S2468-1253(18)30161-4
2. K.Jesuratnam-Nielsen, Løgager VB, Rezanavaz-Gheshlagh B, et al. (2015) Plain magnetic resonance imaging as an alternative in evaluating inflammation and bowel damage in inflammatory bowel disease - A prospective comparison with conventional magnetic resonance follow-through. Scand J Gastroenterol. doi: 10.3109/00365521.2014.1003398 LK
3. Bruining DH, Bhatnagar G, Rimola J, et al. (2015) CT and MR enterography in Crohn's disease: current and future applications. Abdom Imaging. doi: 10.1007/s00261-015-0360-9
4. Laghi A, Paolantonio P, Iafrate F, et al. MR of the small bowel with a biphasic oral contrast agent (polyethylene glycol): technical aspects and findings in patients affected by Crohn's disease. Radiol medica 106:18–27.
5. Ajaj W, Goehde SC, Schneemann H, et al. (2004) Dose optimization of mannitol solution for small bowel distension in MRI. J Magn Reson Imaging 20:648–653. doi: 10.1002/jmri.20166
6. Maccioni F, Viscido A, Marini M, Caprilli R (2002) MRI evaluation of Crohn's disease of the small and large bowel with the use of negative superparamagnetic oral contrast agents. Abdom Imaging. doi: 10.1007/s00261-001-0119-3

7. Ajaj W, Goehde SC, Schneemann H, et al. (2004) Oral contrast agent for small bowel MRI: Comparison of different additives to optimize bowel distension. Eur Radiol. doi: 10.1007/s00330-003-2177-0

8. Taylor SA, Avni F, Cronin CG, Hoeffel c, Kim SH, Laghi A, Napolitano M, Petit P, Rimola J, Tolan DJ, Torkzad MR, Zappa M, Bhatnagar G, Puylaert CAJ SJ (2017) The first joint ESGAR/ ESPR consensus statement on the technical performance of cross-sectional small bowel and colonic imaging. Eur Radiol 27:2570–2582. doi: 10.1007/s00330-016-4615-9

9. Miles A, Bhatnagar G, Halligan S, et al. (2018) Magnetic resonance enterography, small bowel ultrasound and colonoscopy to diagnose and stage Crohn's disease: patient acceptability and perceived burden. Eur Radiol 43–45. doi: 10.1007/s00330-018-5661-2

10. Young BM, Fletcher JG, Booya F, et al. (2008) Head-to-head comparison of oral contrast agents for cross-sectional enterography: Small bowel distention, timing, and side effects. J Comput Assist Tomogr 32:32–38. doi: 10.1097/RCT.0b013e318061961d

11. Sood RR, Joubert I, Franklin H, et al. (2002) Small bowel MRI: Comparison of a polyethylene glycol preparation and water as oral contrast media. J Magn Reson Imaging 15:401–408. doi: 10.1002/jmri.10090

12. Lauenstein TC, Schneemann H, Vogt FM, et al. (2003) Optimization of Oral Contrast Agents for MR Imaging of the Small Bowel 1. Radiology 228:279–283.

13. Kuehle C a, Ajaj W, Ladd SC, et al. (2006) Hydro-MRI of the small bowel: effect of contrast volume, timing of contrast administration, and data acquisition on bowel distention. AJR Am J Roentgenol 187:W375-85. doi:

10.2214/AJR.05.1079

14. Kinner S, Kuehle CA, Herbig S, et al. (2008) MRI of the small bowel: Can sufficient bowel distension be achieved with small volumes of oral contrast? Eur Radiol 18:2542–2548. doi: 10.1007/s00330-008-1041-7

15. Ajaj W, Goyen M, Schneemann H, et al. (2005) Oral contrast agents for small bowel distension in MRI: Influence of the osmolarity for small bowel distention. Eur Radiol 15:1400–1406. doi: 10.1007/s00330-005-2711-3

16. Laghi, Andrea, Pasquale Paolantonio, Franco Iafrate, Osvaldo Borrelli, Lucia Dito, Ernesto Tomei, Salvatore Cucchiara and RP MR of the Small Bowel with a Biphasic Oral Contrast Agent (polyethylene Glycol): Technical Aspects and Findings in Patients Affected by Crohn's Disease. Radiol. Medica

17. Absah I, Bruining DH, Matsumoto JM, et al. (2012) MR enterography in pediatric inflammatory bowel disease: Retrospective assessment of patient tolerance, image quality, and initial performance estimates. Am J Roentgenol 199:367–375. doi: 10.2214/AJR.11.8363

18. Gottumukkala R V., LaPointe A, Sargent D, Gee MS (2019) Comparison of three oral contrast preparations for magnetic resonance enterography in pediatric patients with known or suspected Crohn disease: a prospective randomized trial. Pediatr Radiol. doi: 10.1007/s00247-019-04378-5

19. Taylor S, Mallett S, Bhatnagar G, et al. (2014) METRIC (MREnterography or ulTRasound in Crohn's disease): a study protocol for a multicentre, non-randomised, single-arm, prospective comparison study of magnetic resonance enterography and small bowel ultrasound compared to a reference standard in those . BMC Gastroenterol 14:142. doi:

10.1186/1471-230X-14-142

20. Taylor SA, Mallett S, Bhatnagar G, et al. (2019) Magnetic resonance enterography compared with ultrasonography in newly diagnosed and relapsing crohn's disease patients: The METRIC diagnostic accuracy study. Health Technol Assess (Rockv) 23:vii–161. doi: 10.3310/hta23420

21. Taylor SA, Halligan S, Goh V, et al. (2003) Optimizing Colonic Distention for Multi – Detector Row CT Colonography : Effect of Hyoscine Butylbromide and Rectal Balloon Catheter 1. Radiology 229:99–108.

22. Saini S, Colak E, Anthwal S, et al. (2014) Comparison of 3% sorbitol vs psyllium fibre as oral contrast agents in MR enterography. Br J Radiol. doi: 10.1259/bjr.20140100

23. Gwet K (2014) Handbook of inter-rater reliability fourth edition.

24. Landis J, Koch G (1977) The measurement of observer agreement for categorical data. Biometrics Mar:159–74.

25. Bekendam MIJ, Puylaert CAJ, Phoa SKSS, et al. (2017) Shortened oral contrast preparation for improved small bowel distension at MR enterography. Abdom Radiol. doi: 10.1007/s00261-017-1133-4

26. Wong J, Moore H, Roger M, McKee C (2016) CT enterography: Mannitol versus VoLumen. J Med Imaging Radiat Oncol 60:593–598. doi: 10.1111/1754-9485.12486

27. Zheng MQ, Zeng QS, Yu YQ, et al. (2020) Evaluation of the performance of two neutral oral contrast agents in computed tomography enterography: A randomized controlled trial. J Dig Dis 21:112–119. doi: 10.1111/1751-2980.12835

Table 1: Number of patients\segments achieving good (or excellent) distension according to oral contrast agent

	Number of patients/segments where distension was graded as excellent/good by at least one observer		
	**Mannitol (n=68) n/N* (%)**	**Polyethylene Glycol (PEG) (n=37) n/N* (%)**	**Difference between contrasts** % (95% CI)**
Patients	37/68 (54)	17/37 (46)	8 (-11 to 28)
Segments			
Duodenum	11/68 (16)	5/37 (14)	2 (-11 to 17)
Jejunum	27/68 (40)	5/37 (14)	26 (10 to 42) p=0.0053
Ileum	51/68 (75)	28/37 (76)	-1 (-18 to 17)
Terminal Ileum	37/68 (54)	20/37 (54)	0 (-20 to 20)
Caecum	22/51 (43)	14/28 (50)	-7 (-30 to 16)
Ascending colon	43/61 (70)	23/33 (70)	0 (-19 to 20)
Transverse colon	41/64 (64)	16/37 (43)	20 (0 to 41) p=0.0420

*In some patients segments had been excised and so could not be assessed.
** Mannitol minus Polyethylene Glycol (PEG)

Table 2: Number of patients/segments achieving good or excellent distension according to mannitol oral contrast volume ingested

	**Volume less or equal than 1L N* (%)**	**Volume more than 1L N* (%)**	**Difference** % (95% CI)**
Patients	17/33 (52)	18/32 (56)	-5 (-29 to 19)
Segments			
Duodenum	5/33 (15)	5/32 (16)	
Jejunum	11/33 (33)	15/32 (47)	-14 (-37 to 10)
Ileum	24/33 (73)	25/32 (78)	
Terminal_ileum	17/33 (52)	19/32 (59)	
Caecum	8/19 (42)	12/30 (40)	
Ascending_C	18/27 (67)	23/31 (74)	
Transverse_C	17/29 (59)	23/32 (72)	-13 (-37 to 10)

*In some patients segments had been excised and so could not be assessed.
** Volume less or equal than 1L vs Volume more than 1L (The statistical power is limited and therefore unable to show a statistical difference between proportions)

Table 3: Excellent or good distension overall and by segment for the two readers

		Reader 1	Reader 2	Agreement	Gwet's AC
Segment	N*	N (%)	N (%)	% (95% CI)	
Overall	105	48 (45)	45 (42)	82 (75 to 90)	0.65
Duodenum	105	11 (10)	9 (8)	87 (80 to 93)	0.84
Jejunum	105	21 (20)	22 (21)	79 (71 to 86)	0.68
Ileum	105	63 (59)	70 (65)	73 (64 to 81)	0.49
Terminal_ileum	105	52 (49)	30 (28)	68 (59 to 77)	0.40
Caecum	80	30 (37)	31 (39)	84 (75 to 92)	0.69
Ascending_C	94	61 (64)	53 (55)	77 (69 to 86)	0.56
Transverse_C	101	53 (51)	33 (32)	69 (60 to 78)	0.40

*In some patients segments had been excised and so could not be assessed.

Table 4: Patient symptoms by contrast agent and time point following MRE

	Mannitol (N=85)			Klean Prep (N=45)		
	Very tolerable	Moderately tolerable	Not tolerable	Very tolerable	Moderately tolerable	Not tolerable
	n/N[a] (%)	n/N[a] (%)	n/N[a] (%)	n/N[a] (%)	n/N[a] (%)	n/N[a] (%)
Symptoms immediately following MRE						
A feeling of fullness	37/84 (44*)	46/84 (55)	1/84 (1)	12/45 (27*)	32/45 (71)	1/45 (2)
Regurgitation	62/83 (75)	18/83 (22)	3/83 (4)	31/43 (72)	8/43 (19)	4/43 (9)
Vomiting	72/81 (89)	7/81 (9)	2/81 (2)	38/44 (86)	3/44 (7)	3/44 (7)
Abdominal pain/ spasms	50/82 (61)	28/82 (34)	4/82 (5)	25/43 (58)	14/43 (33)	4/43 (9)
Diarrhoea	54/82 (66)	24/82 (29)	4/82 (5)	25/44 (57)	12/44 (27)	7/44 (16)
Symptoms for the 2 days following MRE						
Flatulence	23/48 (48)	20/48 (42)	5/48 (10)	22/42 (52)	15/42 (36)	5/42 (12)
Regurgitation	39/43 (91)	4/43 (9)	0/43 (0)	33/41 (80)	6/41 (15)	2/41 (5)
Vomiting	39/44 (89)	3/44 (7)	2/44 (5)	36/41 (88)	3/41 (7)	2/41 (5)
Abdominal pain/ spasms	15/49 (31)	29/49 (59)	5/49 (10)	18/42 (43)	19/42 (45)	5/42 (12)
Diarrhoea	24/55 (44)	22/55 (40)	9/55 (16)	14/44 (32)	24/44 (55)	6/44 (14)

a The number of patients answering each survey question varies, so numbers and percentages are given for those responding.

*The only significant difference in experience between patients receiving mannitol and those receiving Klean Prep was in the feeling of fullness immediately after contrast, where 17% more patients (95% CI 0.6 to 34%) had a very tolerable feeling of fullness with mannitol compared to Klean Prep.

Table 5: Patient experience according to mannitol oral contrast volume ingested

	Volume less or equal than 1L (N=40)			Volume more than 1L (N=45)		
	Very tolerable	Moderately tolerable	Not tolerable	Very tolerable	Moderately tolerable	Not tolerable
	n/N[a] (%)	n/N[a] (%)	n/N[a] (%)	n/N[a] (%)	n/N[a] (%)	n/N[a] (%)
Symptoms immediately following MRE						
A feeling of fullness	18/39 (46)	20/39 (51)	1/39 (3)	19/45 (42)	26/45 (58)	0/45 (0)
Regurgitation	30/40 (75)	10/40 (25)	0/40 (0)	32/43 (74)	8/43 (19)	3/43 (7)
Vomiting	34/38 (89)	4/38 (11)	0/38 (0)	38/43 (88)	3/43 (7)	2/43 (5)
Abdominal pain/ spasms	25/39 (64)	13/39 (33)	1/39 (3)	25/43 (58)	15/43 (35)	3/43 (7)
Diarrhoea	23/39 (59)	14/39 (36)	2/39 (5)	31/43 (72)	10/43 (23)	2/43 (5)
Symptoms up to 2 days following MRE						
Flatulence	14/26 (54)	12/26 (46)	0/26 (0)	9/22 (41)	8/22 (36)	5/22 (23)
Regurgitation	23/24 (96)	1/24 (4)	0/24 (0)	16/19 (84)	3/19 (16)	0/19 (0)
Vomiting	23/25 (92)	2/25 (8)	0/25 (0)	16/19 (84)	1/19 (5)	2/19 (11)
Abdominal pain/ spasms	9/26 (35)	14/26 (54)	3/26 (12)	6/23 (26)	15/23 (65)	2/23 (9)
Diarrhoea	14/30 (47)	11/30 (37)	5/30 (17)	10/25 (40)	11/25 (44)	4/25 (16)

The number of patients answering each survey question varies, so numbers and percentages are given for those responding.

Figure 1: Comparison of patient symptoms dependent on oral preparation ((a)mannitol based and (b) PEG based) immediately after and up to two days after the MRE

Figure 1a

Click here to access/download;Figure;Figure 1a

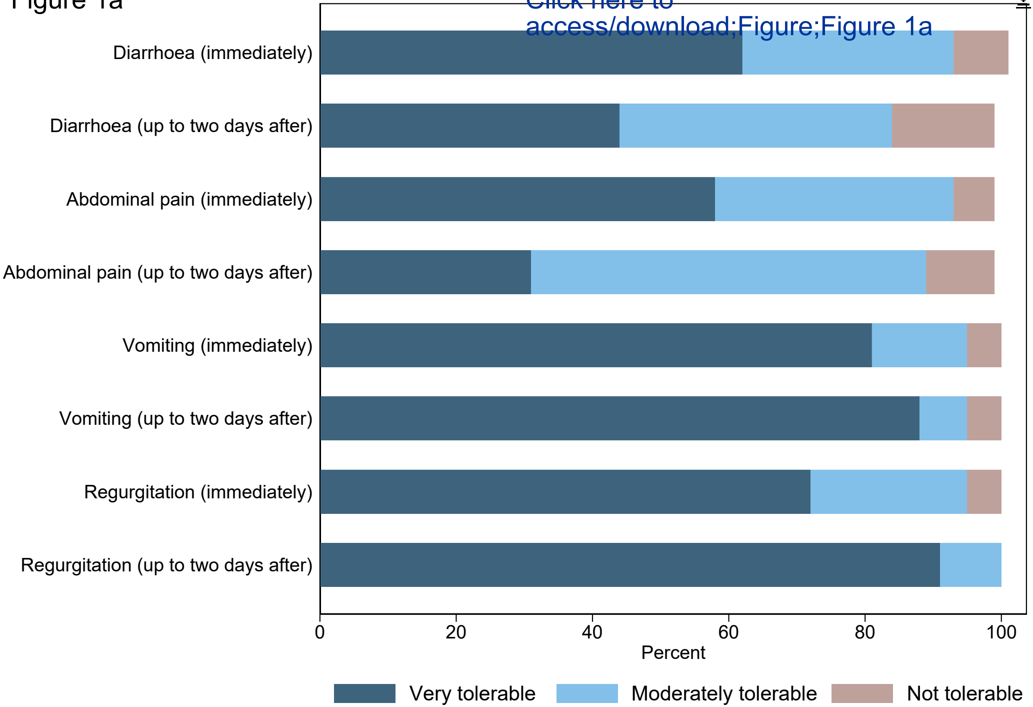

Figure 1b

Click here to access/download;Figure;Figure 1b

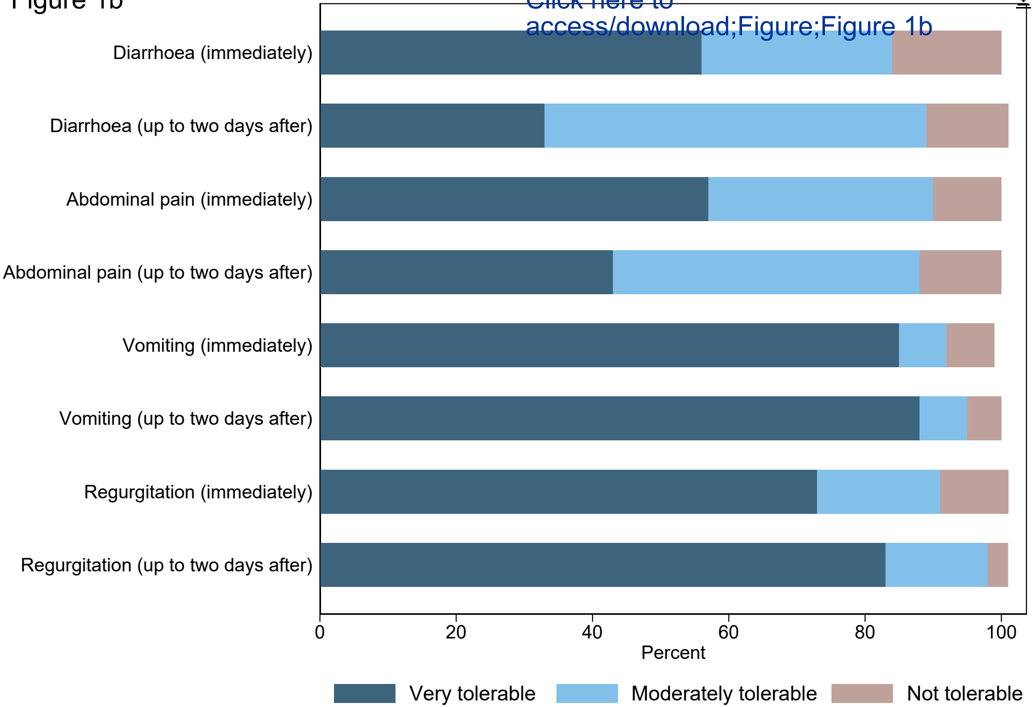

Click here to access/download;Disclosure Paragraph;EURA-D-21-04028_disclosure paragraph.docx

Funding
This study has received funding by NIHR HTA 11/23/01 and the UCLH NIHR BRC.

Guarantor:
The scientific guarantor of this publication is Professor Stuart A Taylor.

Conflict of Interest:
The authors of this manuscript declare no relationships with any companies whose products or services may be related to the subject matter of the article.

Statistics and Biometry:
Professor Sue Mallett kindly provided statistical advice for this manuscript.

Informed Consent:
Written informed consent was obtained from all subjects (patients) in this study.

Ethical Approval:
Institutional Review Board approval was obtained.

Study subjects or cohorts overlap:
A subset of 66 patients from this study have been previously reported in Taylor SA, Mallett S, Bhatnagar G, et al. (2019) Magnetic resonance enterography compared with ultrasonography in newly diagnosed and relapsing crohn's disease patients: The METRIC diagnostic accuracy study. Health Technol Assess (Rockv) 23:vii–161. doi: 10.3310/hta23420.

Methodology
- prospective
- observational
- multicenter study

Click here to access/download
Supplementary Material
EURA-D-21-04028_supplementary material.docx

Click here to access/download
Supplementary Material
EURA-D-21-04028_appendix 2.pdf

Title Page (Title, Authors, Institutions, Contact Information)

Click here to access/download
Not for review
Title page.docx

Main Document (no author names)

Click here to access/download
Not for review
BLIND revised Manuscript with comments.docx

Click here to access/download
Not for review
Disclosure paragraph.docx

Main Document (no author names)

Click here to access/download
Not for review
BLIND revised Manuscript clean.docx

Supplementary Appendix 2

Click here to access/download

Not for review

Appendix 2 BLIND CRF .pdf

Supplementary Appendix 4

Click here to access/download

Not for review

Appendix 4 Figure 2.pdf

Click here to access/download

Not for review

Appendix 6 Figure 3.pdf

Click here to access/download
Not for review
Appendices clean version.docx
